# Modelling innovation performance of European regions using multi-output neural networks

**DOI:** 10.1371/journal.pone.0185755

**Published:** 2017-10-02

**Authors:** Petr Hajek, Roberto Henriques

**Affiliations:** 1 Institute of System Engineering and Informatics, Faculty of Economics and Administration, University of Pardubice, Studentská 84, Pardubice, Czech Republic; 2 ISEGI, Universidade Nova de Lisboa, Lisboa, Portugal; Universidad de Castilla-La Mancha, SPAIN

## Abstract

Regional innovation performance is an important indicator for decision-making regarding the implementation of policies intended to support innovation. However, patterns in regional innovation structures are becoming increasingly diverse, complex and nonlinear. To address these issues, this study aims to develop a model based on a multi-output neural network. Both intra- and inter-regional determinants of innovation performance are empirically investigated using data from the 4^th^ and 5^th^ Community Innovation Surveys of NUTS 2 (Nomenclature of Territorial Units for Statistics) regions. The results suggest that specific innovation strategies must be developed based on the current state of input attributes in the region. Thus, it is possible to develop appropriate strategies and targeted interventions to improve regional innovation performance. We demonstrate that support of entrepreneurship is an effective instrument of innovation policy. We also provide empirical support that both business and government R&D activity have a sigmoidal effect, implying that the most effective R&D support should be directed to regions with below-average and average R&D activity. We further show that the multi-output neural network outperforms traditional statistical and machine learning regression models. In general, therefore, it seems that the proposed model can effectively reflect both the multiple-output nature of innovation performance and the interdependency of the output attributes.

## Introduction

Innovation has been recognized as a central issue in EU development and one of the crucial drivers of economic welfare, especially since the introduction of the Lisbon strategy. Regions in the EU are important since they profit from spatial proximity and knowledge spillovers. Thus, effective formal and informal cooperation among regional actors (both within companies as well as between firms and other organizations such as universities, innovation centers, educational institutions, etc.) can be established. According to [1], cooperation is considered as an innovation stimulus providing benefits such as achieving economies of scale and of scope, reducing uncertainty, gaining access to new markets or accessing complementary knowledge. These interactions may lead to the emergence of a regional innovation system (RIS) [2].

Recently, this concept of the RIS has received increased attention from both policy-makers and academic researchers, expanding our understanding of the innovation process in the regional dimension [3,4]. The RIS literature defines a region as “a territory less than its sovereign state, possessing distinctive supra-local administrative, cultural, political, or economic power, differentiating it from its state and other regions” [2]. Doloreux [5] defines an RIS as a set of interacting private and public interests, formal institutions and other organizations that function according to organizational and institutional arrangements and relationships conducive to the generation, use and dissemination of knowledge. In RISs, intensive communication and cooperation between different actors, such as firms and other organizations (e.g., educational and research institutions), takes place [6].

The research to date has tended to focus on firm-level innovation performance [7–12]. Region-specific characteristics have been used only as the determinants of firm innovation performance in this previous research, without considering the effect of research and innovation policies on the innovation performance of the whole region. Moreover, the regional level is also considered critical for the design and implementation of innovation policies, as regions create appropriate contexts for knowledge creation and transfer [4]. In fact, European research and innovation policies are carried out at the level of regions, rather than at the level of the Member states. An effective research and innovation policy therefore requires modelling innovation performance at the regional level.

Regional (and national) innovation performance is annually measured in the EU using the innovation scoreboard, which captures almost thirty innovation indicators classified into various innovation dimensions [13]. Previous studies have suggested that regional innovation performance cannot be reliably measured using a single indicator. On the contrary, its measurement rather requires a combination of strongly related indicators [3,4], including, among others, the numbers of innovators and the economic effects of innovation activity. However, no previous research known to us has used the multiple-output character of regional innovation performance to examine its determinants. Here, we fill this gap, developing a multi-input multi-output model that also accounts for nonlinear input-output effects. Moreover, the linear models of innovation process (‘‘market pull” or ‘‘technology push” models) were insufficient to induce the transfer of knowledge and technology [14]. Therefore, nonlinear models of innovation have been introduced to take interactive and recursive terms into account. Nonlinear models, such as artificial neural networks (ANNs) [15] or adaptive neuro-fuzzy inference systems [16], have shown promising results in modeling the innovation patterns of firms embedded in regional innovation structures. For the case of Taiwanese firms’ innovation performance, Wang and Chien [15] demonstrated the dominance of ANN models over traditional statistical regression models regarding accuracy. ANNs have also been reported to accurately predict the technology implementation performance of firms [17] and R&D performance of European countries [18]. Additionally, ANNs preserve the experimental knowledge, enable their further use, and are robust to noise (irrelevant data, outliers, etc.). In this study, we argue that multi-output ANN model is more appropriate to model intrinsic multi-output and nonlinear character of innovation performance than traditional statistical and machine learning regression models. Specifically, the previously applied regression models were based on the assumption that the innovation data is best presented linearly. However, recent empirical evidence suggests that the relationships between innovation performance and its determinants are more complex than considered previously, including sigmoidal and inverted U-turned effects of R&D expenditures and cooperation, respectively [9]. Moreover, model specification is required in conventional regression models and it is extremely difficult to combine two models in the same regression model [19]. The specification of these models is also considered not adaptive and require knowledge of the underlying data distribution. As a consequence, conventional regression models can be distorted in evolving, noisy and high variance environments. In contrast, ANNs can automatically represent highly complex input-output non-linear relationships. At the same time, linear patterns are not ignored, and fixed and random effects are incorporated. The ANN model also does not require explicit specification of a priori relationships between input and output attributes. The connectionist architecture and distributed computations in ANNs ensure robustness even when model assumptions are violated.

Previous literature regarding innovation performance modeling has tended to focus on firm-related determinants, paying little attention to the regional framework. Our model gives particular attention to external (to the firm) determinants of innovation performance. The complex region-related determinants of innovation performance lead to an even more complex modeling problem [20]. The aim of this paper is to adopt a regional perspective on the sources of innovation performance. Specifically, here we show that both business and government R&D activity has a positive effect on regional innovation performance. These effects are represented by sigmoidal curves. The nonlinear character of these effects has important implications for regional innovation policy.

The remainder of this paper is structured as follows. In the next section, we operationalize the determinants of regional innovation performance and devise the forecasting model. Section 3 provides the experimental results. Finally, we discuss the obtained results, draw some political implications and conclude the paper with suggestions for future research.

## Materials and methods

### Model and variable description

#### Determinants of regional innovation performance

Two inter-related categories of determinants have been introduced into the models of regional innovation performance, namely internal (firm-specific) and external determinants of innovation. Firm-specific determinants usually include firm R&D expenditure, linkages, and throughputs [21,22]. External determinants can be further subdivided into three categories [23]: (1) the National Innovation System (NIS) and national R&D infrastructure; (2) regional determinants (e.g., local endowment, social capital, regional linkages); and (3) sectoral determinants (e.g., technology, finance, market). Samara et al. [24] provided an alternative categorization of interacting subsystems, including knowledge and human resources, economic and institutional conditions and the financial subsystem (public R&D expenditure and venture capital availability).

**Fig 1:**
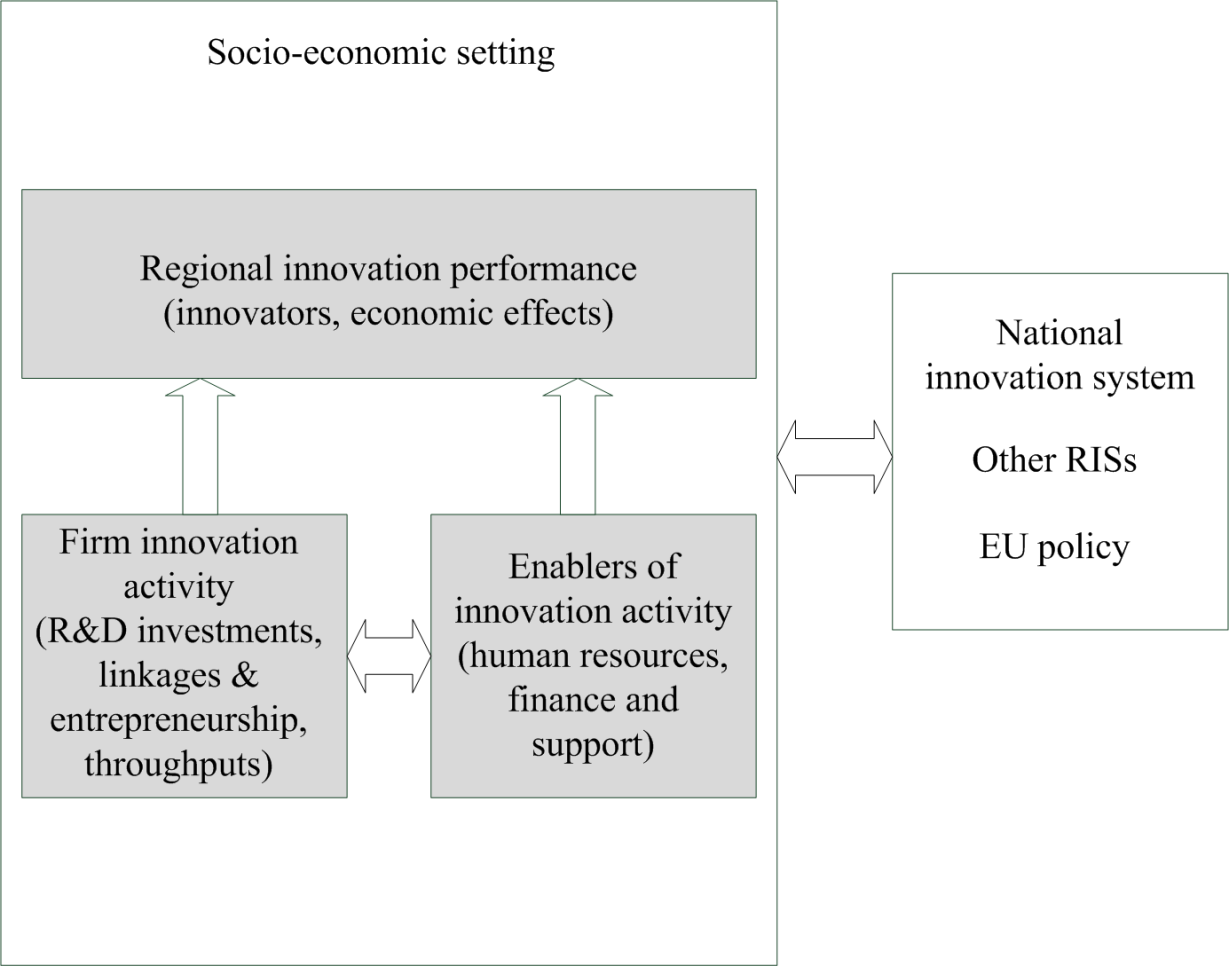
Model of regional innovation system with intra-regional and inter-regional components. Regional innovation performance is affected by internal (firm-specific) and external determinants of innovation (inter-regional component, socioeconomic setting, and enablers of innovation activity).

The functional approach used here is based on previous experimental work [6,24]. It includes two interconnected components: intra-regional and inter-regional. The RIS, conceptualized in Fig 1, can be characterized by a high intensity of economic and knowledge interaction, both within companies as well as between firms and other organizations, such as universities and research centers. These interactions are facilitated by economic and socio-cultural factors, such as routines, shared values, norms, and trust [25]. Thus, the borders of the system can be determined by the frequency or intensity of these interactions.

In this study, we used the determinants and indicators of regional innovation performance presented in Table 1 along with the sources for each type of data. Note that attributes *x*_33_ and *x*_42_ were collected at the level of European nations, while the CIS 4 and CIS 5 firm-level data were aggregated at the regional level to achieve data consistency and applicability to European research and innovation policies.

**Table 1:** Input and output attributes for regional innovation performance forecasting.

	**Input attributes**		
	*1. Intra-regional determinants*		
	*1.1 Socio-economic setting*	Source of data	Level of data
*x*_1_	GDP at current market prices per capita	Eurostat	Regional
*x*_2_	Employment rate [%]	Eurostat	Regional
*x*_3_	Long-term unemployment share [%]	Eurostat	Regional
*x*_4_-*x*_9_	Employment in agr., ind., const., services, finance and public sector [%]	Eurostat	Regional
*x*_10_	Herfindahl index of economic diversification	Eurostat	Regional
	*1.2 Enablers*		
	*1.2.1 Human resources*		
*x*_11_	Population aged 15 and over with tertiary education per 1000 inhabitants	Eurostat	Regional
*x*_12_	Population aged 15 and over with secondary education per 1000 inhabitants	Eurostat	Regional
*x*_13_	Participation of adults aged 25–64 in life-long learning per 1000 inhabitants	Eurostat	Regional
*x*_14_	Human resources in science and technology [%]	Eurostat	Regional
	*1.2.2 Finance and support*		
*x*_15_	Government R&D expenditure in percentage of GDP	Eurostat	Regional
*x*_16_	Higher education R&D expenditure in percentage of GDP	Eurostat	Regional
*x*_17_	Broadband access by households	Eurostat	Regional
*x*_18_	New foreign firms per million inhabitants	ISLA-Bocconi	Regional
*x*_19_	Venture capital relative to GDP	EVCA	National
*x*_20_	Motorway density	Eurostat	Regional
	*1.3 Firm innovation activity*		
	*1.3.1 R&D investments*		
*x*_21_	Business R&D expenditure in percentage of GDP [%]	Eurostat	Regional
*x*_22_	Business non-R&D expenditure in percentage of total turnover [%]	Eurostat (CIS 4)	Firm->Regional
*x*_23_	SMEs innovating in-house [%]	Eurostat (CIS 4)	Firm->Regional
	*1.3.2 Linkages & entrepreneurship*		
*x*_24_	Collaboration on innovations–breadth	Eurostat (CIS 4)	Firm->Regional
*x*_25_	Collaboration on innovations–depth	Eurostat (CIS 4)	Firm->Regional
*x*_26_	Global pipelines	Eurostat (CIS 4)	Firm->Regional
*x*_27_	Knowledge sources–breadth	Eurostat (CIS 4)	Firm->Regional
*x*_28_	Knowledge sources–depth	Eurostat (CIS 4)	Firm->Regional
*x*_29_	Self-employed persons per 1000 inhabitants	Eurostat	Regional
	*1.3.3 Throughputs*		
*x*_30_	High-technology patent applications to the EPO per million of inhabitants	Eurostat	Regional
*x*_31_	Biotechnology patent applications to the EPO per million of inhabitants	Eurostat	Regional
*x*_32_	Patent applications to the EPO per million of inhabitants	Eurostat	Regional
	*2. Inter-regional determinants*		
*x*_33_	Type of national innovation system (nominal attribute: Innovation leaders / Innovation followers / Moderate innovators / Catching-up countries)	Eurostat (CIS 4)	National
*x*_34_- *x*_41_	Knowledge spillovers from neighbouring RISs (*x*_34_—product/process innovators; *x*_35_—marketing/organisational innovators; *x*_36_—new-to-market sales; *x*_37_—new-to-firm sales; *x*_38_—resource efficiency innovators (labour); *x*_39_—resource efficiency innovators (energy); *x*_40_—employment in high-tech manufacturing; *x*_41_—employment in knowledge-intensive services)	Eurostat (CIS 4)	Regional
*x*_42_	EU policy (dummy attribute: new/old Member State)	Eurostat	National
	**Output attributes**		
	*Innovators*		
*y*_1_	Product and/or process innovators [%]	Eurostat (CIS 5)	Firm->Regional
*y*_2_	Marketing and/or organisational innovators [%]	Eurostat (CIS 5)	Firm->Regional
*y*_3_	Resource efficiency innovators—labour [%]	Eurostat (CIS 5)	Firm->Regional
*y*_4_	Resource efficiency innovators—energy [%]	Eurostat (CIS 5)	Firm->Regional
	*Economic effects*		
*y*_5_	New-to-market sales (in percentage of total turnover) [%]	Eurostat (CIS 5)	Firm->Regional
*y*_6_	New-to-firm sales (in percentage of total turnover) [%]	Eurostat (CIS 5)	Firm->Regional

#### Regional socio-economic setting

Regional innovation activity is embedded in a common regional socio-economic setting. According to Cooke [2], differences in socio-economic conditions may lead to divergent innovation performance across regions.

We used regional per capita GDP as a proxy of regional economic performance and technological development [26]. Income per capita has also been employed in several studies as an alternative to GDP [27]. In our study, the economic and market structures were measured by the shares of employment by industry and by the employment Herfindahl index (the degree of market concentration/specialization), respectively. Fritsch and Slavtchev [28] found an inversely U-shaped relationship between regional specialization and R&D efficiency. Also, the economic and market structures, like R&D expenditure, must be incorporated as a potential source of endogeneity. Fritsch and Meschede [22] identified employment structure (especially in knowledge-intensive industries) as another important region-related determinant. De Bruijn and Lagendijk [29] also demonstrated that regional per capita GDP and employment structure are important determinants of RIS performance.

The “social filter” concerns the specific combination of social conditions in a region that determines the ability of the region to transform R&D into innovation [26]. Thus, the social filter becomes an important determinant of long-term regional economic growth [30]. Both employment rate and the share of long-term unemployment have been used as proxies for the social filter. The latter also indicates the rigidity of the regional labor market [26]. Note that the population’s level of education has also been considered an important determinant of the regional social filter [26]. In our study, we adopt the concept of the regional innovation process from the European Innovation Scoreboard [31], in which the population’s level of education is considered to enable innovative activity.

#### Enablers of innovation activity

Enablers of innovation activity include: (1) the availability of human resources (skilled and educated people) and (2) financial sources and other support.

Indicators of human capital can be used to estimate regional capacity to absorb knowledge and technology. We used the share of the population with a secondary education to indicate general skills. Also, the share of the population with a tertiary education was used to assess advanced academic skills [26]. Several studies have also included an index of university quality based on rankings of universities. However, better-ranked universities do not generate significantly higher regional innovation output; therefore, university rankings cannot adequately measure differences in university quality [32].

Human resources in science and technology approximate the demand for and supply of science and technology workers. Sleuwaegen and Boiardi [32] reported that this indicator, together with the share of the population with a tertiary education, represent a major factor (the so-called regional intelligence) of regional innovation activity. The readiness to continue technological development and innovation can be promoted through the participation of people in lifelong learning [27].

In our study, the financial and other support category includes: (1) public R&D expenditure; (2) ICT and transport infrastructure; and (3) foreign direct investment (FDI) and venture capital funds. The level of public R&D expenditure indicates the relative effort of a region to create, disseminate and exploit knowledge. In agreement with previous studies [27], we distinguish between government and higher education R&D expenditures to take into account the different types of research carried out by various public organizations.

ICT and transport infrastructure (communication and transportation networks) make the innovation process more efficient. They also provide accessibility to globally dispersed knowledge sources [32]. By other studies, we used the level of broadband access as a proxy for ICT infrastructure [3] and motorway density as a proxy for transport infrastructure [33].

To estimate the level of FDI, we adopted the approach of Casi and Resmini [34], using the number of new foreign firms per million inhabitants. FDI can contribute significantly to regional innovation capacity and produce positive productivity spillovers [35], but the strength of these effects depends on the availability of the region’s absorptive capacity. Similarly, the positive effect of venture capital varies with the intensity of regional innovation [36]. To measure venture capital funds, we used the indicator of national venture capital relative to GDP obtained from the European Private Equity and Venture Capital Association (EVCA).

#### Firm innovation activities

In this study, firm innovation activities are represented by (1) firm R&D investments; (2) linkages & entrepreneurship; and (3) throughputs.

In-house R&D investment has been recognized as a significant determinant of innovation performance in most studies [37]. This indicator estimates the level of knowledge creation within firms, and it is also associated with the firms’ capacity to absorb more knowledge from the outside. Non-R&D innovation expenditures, on the other hand, measure the diffusion of technology [38]. According to the Frascati Manual of the OECD [39], examining the expenditures of all aspects of the innovation process may facilitate more meaningful calculations of financial inputs. In addition to R&D and non-R&D innovation expenditures, we also used the percentage of SMEs innovating in-house. This indicator has also been used to estimate the transmission and application of knowledge [40].

Firm innovation activities involve the use of both codified (intellectual property rights) and tacit knowledge (through linkages and entrepreneurship). The past decade has seen a rapid rise in firms’ use of the “open innovation” model. A wide range of external actors and knowledge sources helps firms achieve and sustain innovation [41–43]. Frenz and Ietto-Gillies [37] reported that the benefits of cooperation in innovation efforts are realized only when a sufficient number of firms cooperates or when firms cooperate internationally. Moreover, innovation performance degrades with more repeat collaborations [44]. In recent literature, social network analysis of collaborative R&D projects has also been used to model knowledge flows between regions [45]. The number of these linkages was reported to be a significant determinant of innovation throughputs. To account for the presence of network connections, in accordance with Laursen and Salter [41], we captured the strengths of the linkages using four dimensions (two-by-two), namely the breadth and depth of both collaboration and knowledge sources. The extent of cooperation on innovation was measured as the number of connections—collaborating partners (other firms, suppliers, clients, competitors, consultants, universities and government institutes), whereas only simultaneous collaborations between domestic and international partners were considered in estimating the depth of collaboration (on a 0–7 scale). Similarly, the breadth of knowledge sources was calculated as the number of connections—knowledge sources used (market: suppliers, clients, competitors, and consultants; institutional: universities and government research institutes; other: conferences, scientific journals, and professional associations). To determine the depth of knowledge sources, only those sources were included that were used to a high degree. To further distinguish between local and global knowledge exchange and to recognize the increasing importance of global pipelines [25,46], we also included the number of connections—collaborating international partners.

The level of entrepreneurial activity relates to the regional capacity to apply new knowledge. It is therefore considered an important determinant of innovation-based regional growth [47]. We used the share of self-employed persons as a proxy for entrepreneurial activity.

In line with the Frascati Manual [39], the numbers of patent applications were considered as throughputs (indicators of intervention) and not as indicators of innovation output. This is for several reasons. First, patent applications do not necessarily lead to innovation. Second, some types of technology are not patentable. To consider the variety of R&D outputs, we discriminated between patent applications in high technology, biotechnology (due to their strong impact on chemical, pharmaceutical, and related research-intensive industries) and all fields considered in total.

#### Inter-regional determinants

The inter-regional determinants of EU regions’ regional innovation performance include the specifics of the corresponding NIS [48], inter-regional knowledge spillovers, and support from the EU.

NISs are important in determining inter-regional disparities [23]. The importance of NISs also stems from the public-private nature of the regional innovation process. To obtain a typology of NISs, we used the summary innovation index from the EIS 2004. This index provides both an aggregate indicator of national innovation performance and outlines the strengths and weaknesses of NISs [49]. To consider the effect of national innovation policy, we categorized NISs based on the four innovation patterns found in the EIS: (1) innovation leaders, (2) innovation followers, (3) moderate innovators, and (4) catching-up countries. Thus, a variety of national-level determinants was considered as the EIS is calculated based on the national indicators of human resources, research systems, innovation-friendly environment, finance & support, firm investments, innovators, linkages, intellectual assets and employment and sales impacts. Previous studies have also included the availability of investment capital as an indicator associated with the national environment [21]. Note that we also used the availability of national venture capital within the enablers of innovation activity. Alternative measures of NISs’ performance have also been considered in previous literature [32,49]. However, most of these evaluate the overall competitive capacity of economic systems, whereas the EIS was conceived specifically as a tool to monitor the objectives of the Lisbon Strategy.

Reportedly, the innovation performance of a region is dependent on the performance of spatially close regions. The strength of this relationship may vary depending on the range of knowledge diffusion [50]. Rodriguez-Pose and Crescenzi [26] found a strong distance decay effect for this relationship, emphasizing the effect of innovative activities pursued in neighboring regions. Similarly, Vila *et al*. [51] indicated that the level of innovation outputs strongly depends on the corresponding outputs in neighboring regions. Specifically, it was empirically confirmed that knowledge spillovers reinforce the effect of local innovative activities as the amount of knowledge available in the neighboring region increases. Moreover, a strong innovative performance in neighboring regions may even compensate for a weak innovation activity pursued locally [26]. Thus, a region within an innovative neighborhood is more advantaged compared with one in less innovative neighborhood. Therefore, we estimated these spillovers using those neighboring regions with the best innovation performance. The size of the knowledge spillover from neighboring RISs was estimated using the following formula:

$$x_{j}=max(y^{k,2004})-y^{2004},$$ (1)

where *j* = 34, 35, …, 39, *k* denotes the index of a neighboring region, and *y*^*2004*^ is the indicator of innovation performance in the given region in 2004, with *y*^*2004*^ = *y*_*1*_^*2004*^, *y*_*2*_^*2004*^, …, *y*_*6*_^*2004*^. The same formula was used to calculate the additional knowledge spillovers *x*_*40*_ and *x*_*41*_ stemming from employment growth in neighboring RISs (*x*_*40*_ for high-technology manufacturing and *x*_*41*_ for knowledge-intensive high-technology services). This is, we assumed that the regions were interconnected in eight dimensions, through product/process innovators, marketing/organizational innovators, new-to-market and new-to-firm sales, resource efficiency innovators (labor and energy), and employment in high-tech manufacturing and knowledge-intensive services. Note that the knowledge spillovers were considered higher with the increasing gap in innovation performance between the given and neighboring regions. This is in agreement with most studies, considering extra-regional knowledge sourcing as complementary to knowledge available regionally [10]. In other words, we abstracted from all other connections among the regions and, thus, the ANN model accounts only for the interdependence of geographically neighboring observations. This was not only due to the strong distance decay effect reported in the literature but also to control for the complexity of the ANN model.

International trade has also been considered an important source of regional knowledge spillovers [52]. However, related data are available only for a handful of EU countries. This study also considered the effect of EU institutional and financial support of RISs, distinguishing between old and the new Member States. EU regional support has shown a significantly positive effect on GDP growth in EU regions [53], strongly contributing to the economic convergence of new Member States [3].

#### Regional innovation output

In the EIS, the output of regional innovation activity was measured by the number of innovators, considering the diversity of innovations. R&D may be the most important innovation determinant for some firms, while other firms may rely on adopting technologies. Thus, it is not possible to use a single indicator of innovation performance. Therefore, several dimensions of innovation performance have been designed by the European Commission, based on the Frascati Manual of the OECD [39], to accommodate the diversity of different innovation processes and models that occur in different regional contexts. The use of different types and modes of innovations in a model leads to better understanding of the drivers of regional innovation performance. In our set of innovation performance indicators, we adopted the approach of the EIS and calculated the proportion of innovative firms in the region, which corresponds to the outputs of innovation generators [54]. Regional innovation output can be measured separately for product or process innovations and for marketing or organizational innovations. The former includes technological innovation, whereas the latter represents non-technological innovation. Furthermore, the latter is an important determinant of productivity growth and the efficiency of product or process innovations. Additionally, technological innovations have an important effect on the efficiency of resource use. Therefore, we also investigated the share of innovating firms that reduced labor costs and materials and energy, respectively, as a result of innovation activity. In addition to the outputs of innovation generators, the EIS evaluates the economic success of innovation activity. Therefore, we also considered the economic benefits (measured as sales) of regional innovation activity. Specifically, we differentiated between new-to-market (the creation of state-of-the-art technologies) and new-to-firm (the diffusion of technologies) sales. Recently, innovation efficiency has been used as an alternative measure to the outputs of innovation generators. In this approach, innovation performance is defined as the relation between the generated innovative output and the invested efforts. However, several strong assumptions must be made to apply this approach (either parametric or non-parametric), for example, the multiplicative effect of innovation facilitators, correct innovation production function, the identity assumption and accurately considered fluctuation [54].

### Data preprocessing

We investigated regional innovation performance at the level of NUTS 2 (Nomenclature of Territorial Units for Statistics) regions, using the baseline NUTS 2 structure from 2006 (271 regions in total). The NUTS classification is based primarily on the institutional divisions, this is on normative criteria. Above all, it is the size of population (0.8–3 million) necessary to efficiently and economically carry out tasks allocated to territorial communities. Historical and cultural factors represent other normative criteria used for the classification. This was introduced for the purpose of socio-economic analyses of the regions. The NUTS 2 are basic regions for the application of regional policies. Most importantly, this level is becoming increasingly important to decision-makers considering the application of R&D and innovation policies. Compared to countries, the NUTS 2 regions are more homogeneous and better connected within themselves [50]. Data for the determinants of regional innovation performance were collected for the year 2004 and 265 European regions (for 6 regions, the data were not available). Input attributes were derived from the fourth Community Innovation Survey (CIS 4). The CISs offer the most comprehensive data regarding the range of firms surveyed. The effects of the determinants of regional innovation performance are expected to be delayed [37]. Therefore, data measuring regional innovation performance (output attributes) were taken from 2006 (CIS 5). The reference periods for the CIS 4 (and 5) were 2004 and 2006, respectively, and the observation period of each ran from 2002 to 2004 and from 2004 to 2006, respectively. The CIS 5 was carried out using either the same harmonized survey questionnaire or a subset of the CIS 4 questions.

S1 Appendix presents the input and output data. In conformity with regional innovation scoreboard, we noticed considerable diversity in regional innovation performance within countries. Most innovative regions were typically located in NISs’ leaders (Germany, United Kingdom, Sweden, etc.), whereas Eastern and Southern European regions performed worst (Bulgaria, Romania, Poland, Portugal, etc.). In fact, regions performing well in terms of GDP per capita and competitiveness index [55] were also performing well with respect to innovation activity. In particular, more competitive and innovative regions were observed in Northern Europe. This can be partly attributed to high institutional quality, macroeconomic stability, the quality of education and labor market efficiency. Generally, capital regions performed better than non-capital regions. However, imbalanced levels of innovation performance indicators were observed for a significant amount of regions. Also note that, on one hand, the European regions lagged behind those in United States, Japan and South Korea in overall R&D intensity in the reference period. On the other hand, growing R&D investment promoted competitiveness and economic growth in many NUTS 2 regions. As others NUTS 2 regions faced efficiency problems related to the inadequate impact of R&D expenditure, the European Union launched ambitious research and innovation policy reforms during the reference period. However, it is difficult to predict the effects of these reforms due to the complexity of the interrelated components of RISs. The multilayer architecture of ANNs enables to represent various combinations of these components. In other words, the connectivity of the ANN simulates the effects of various regional research and innovation policies. In addition, the number of neurons in ANN layers control for the complexity of these combinations. In fact, a highly complex system can be theoretically designed to model all specifications of the regions in the dataset. However, such a model would be overfitted to the regions in the training dataset, performing poorly on out-of-sample regions. Therefore, we believe that the NUTS 2 regional level represents a natural context to use the ANN approach for innovation performance modelling.

First, missing values in the data had to be treated. If data for surrounding years were available, we replaced missing data using linear regression based on the data of the corresponding NUTS 1 regions. If these data were not available, we replaced the missing data with the NUTS 1 data. During data pre-processing, principal component analysis (PCA) was conducted to obtain a set of uncorrelated variables.

Pakath and Zaveri [56] stated that neural networks are highly dependent on the selection of input variables. In that sense, reducing the dimensions of the input variables will extract the important variables to increase the execution efficiency of the models. Also, learning algorithms operate faster, and accuracy of the models can be improved [57,58]. Feature extraction using the PCA was also used in previous studies on modeling innovation performance to avoid the collinearity among input attributes [15,16].

Twelve components were detected with eigenvalues greater than one. These components explained 73.2% of the total variance in the data. The components were further labeled (Table 2) based on the component loadings presented in S2 Appendix.

**Table 2:** Results of principal component analysis.

	Label (three largest component loadings)	Eigenvalue	Variance (%)
*CP*_1_	Business R&D activity (*x*_32_, *x*_30_, *x*_21_)	11.36	25.82
*CP*_2_	Linkages (*x*_24_, *x*_26_, *x*_27_)	4.26	9.67
*CP*_3_	Human resources—secondary education (*x*_12_, *x*_5_, *x*_42_)	3.37	7.66
*CP*_4_	Economic effects of inter-regional knowledge spillovers (*x*_39_, *x*_37_, *x*_36_)	2.55	5.80
*CP*_5_	Labour-market flexibility (*x*_2_, *x*_13_, *x*_23_)	2.33	5.29
*CP*_6_	Economic diversification (*x*_10_, *x*_9_, *x*_17_)	1.79	4.07
*CP*_7_	Technological and non-technological inter-regional knowledge spillovers (*x*_35_, *x*_34_, *x*_41_)	1.63	3.71
*CP*_8_	Foreign direct investment (*x*_18_, *x*_33c_, *x*_42_)	1.43	3.24
*CP*_9_	Catching-up countries (*x*_4_, *x*_13_, *x*_19_)	1.37	3.11
*CP*_10_	Entrepreneurship (*x*_29_, *x*_22_, *x*_4_)	1.23	2.80
*CP*_11_	Public R&D expenditure (*x*_15_, *x*_40_, *x*_14_)	1.16	2.62
*CP*_12_	Human resources—tertiary education (*x*_11_, *x*_31_, *x*_22_)	1.04	2.36

## Methods

ANNs are an approach for quantitative data analysis inspired by the way we believe the brain processes information. It consists of a processing system composed by a large number of interconnected elements (units or neurons) which are usually organized into a sequence of layers (the input layer, hidden layer(s) and output layer) linked by weights. The networks are then trained by processing the inputs and comparing the resulting outputs with the desired outputs. The difference is then propagated to the network by adjusting its weights to minimize the error. This is an iterative process, and the weights are continually adjusted until it converges to an optimum solution. This learning capability makes them very suitable for different specific applications, such as pattern recognition or data classification [59]. ANNs represent not a single method but a family of models. The ANN model chosen for this research is the Multilayer Perceptron (MLP), i.e., a multi-layer, fully connected feed-forward network with a back-propagation training rule as proposed by [60].

**Fig 2:**
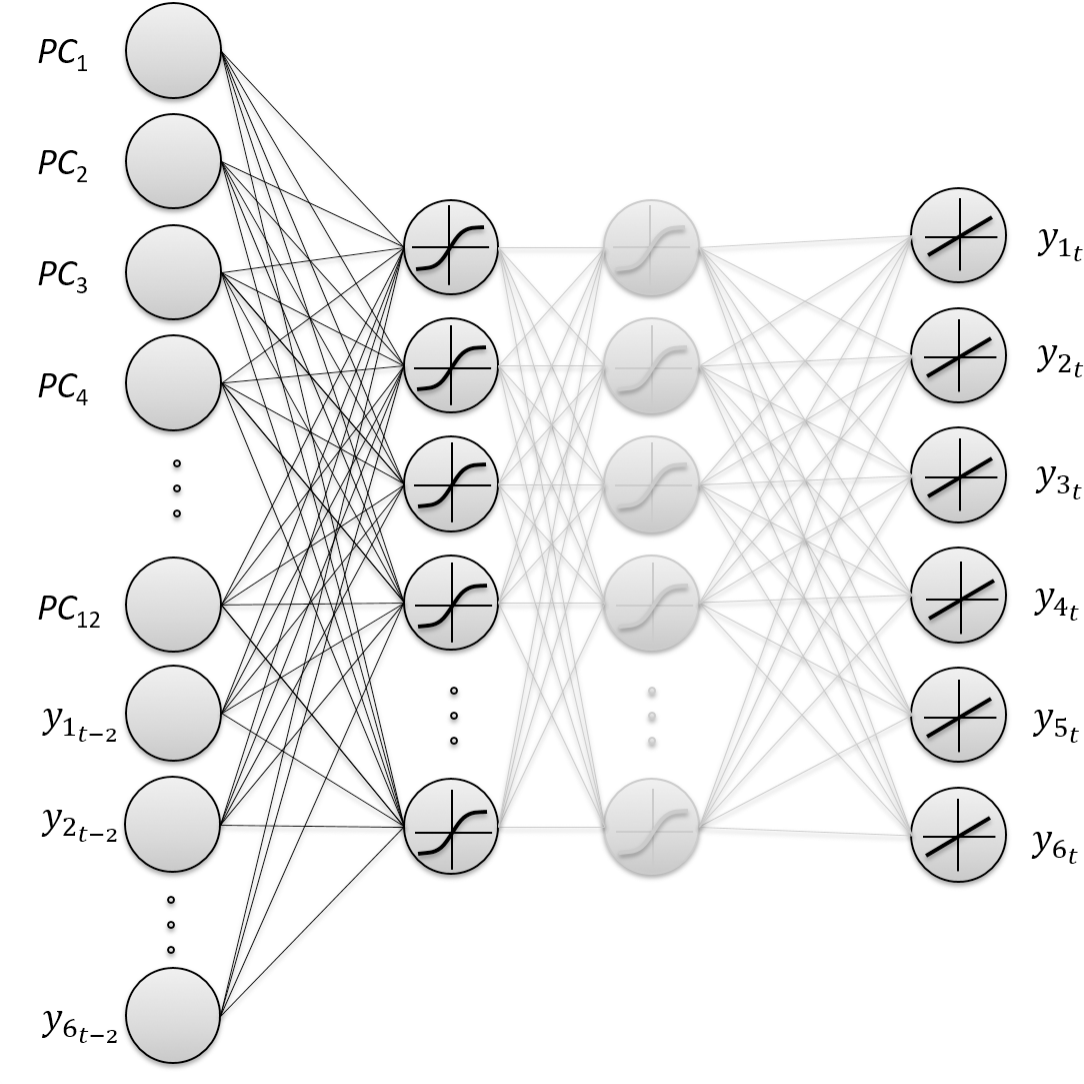
General multi-output MLP architecture. The input layer presents 18 neurons, corresponding to the 12 principal components presented in Table 2 plus six outputs regarding the outputs in time *t-2*. Several MLP architectures were tested by changing the number of layers and the number of neurons in each hidden layer. Neurons in the output layer represent the multi-output regional innovation performance in time *t*.

We employed the MLP to forecast the innovation performance of each NUTS 2 region. MLP comprises fully connected neurons organized into a sequence of layers. The general architecture of the MLP used in this study is depicted in Fig 2. The input layer represents the components of regional innovation performance, the hidden layer represents the combinations of these components (regional innovation policies), and the output layer represents the outputs of regional innovation activity (innovators and their economic effects). The connections between neurons in the MLP denote the importance of the components for regional innovation policies (connections between the input and hidden layer) and the importance of these policies for the outputs of regional innovation performance (connections between the hidden and output layer), respectively. Note that more complex combinations of the regional innovation components can be modelled using the second hidden layer, which can extract high-order regional policies (general and more specific, for example). The sigmoid activation functions of the neurons in the hidden layer enable to find non-linear patterns (combinations of regional innovation components) in the data.

The time-series component of the outputs was considered using six outputs at time *t*-2. However, Qi and Zhang [61] presented empirical evidence that stationarity is important for the out-of-sample performance of the ANNs regardless of the underlying data generating process. Therefore, we first performed the Hadri Lagrange multiplier panel unit root test for a null hypothesis that the individual observed are stationary around a deterministic level or around a deterministic trend [62]. We found clear evidence of non-stationarity for all six outputs. To reduce the effect of nonstationarity, we used the logarithms of outputs in our model. The output layer is formed by six output neurons, corresponding to the six outputs in time t presented in Table 1. The transfer functions selected for the layers were sigmoid for the hidden layer and linear for the output layer. A normalization step (into the range [−1, 1]) was applied to both the input vectors and the target vectors using the mapminmax function. The MLP was trained with the Levenberg–Marquardt [63] back-propagation algorithm in the Matlab Neural Network Toolbox.

The early stopping procedure followed in this paper was defined based on MSE gradient. The training phase will stop if the magnitude of the gradient of MSE for validation data is less than 1e-5 for six successive iterations.

The use of multiple layers and neurons not only enables modeling of nonlinear input-output relationships, but it also facilitates measuring the effects of endogenous variables and their interrelations [64]. This feature is necessary to solve the estimation problems reported in previous literature. For example, R&D expenditure and entrepreneurial activity have been reported as a source of strong endogeneity problems [22]. Moreover, the multi-output ANN model addresses the issue of the possible interdependency of the output attributes. For example, Kraft [65] reported the interdependence of product and process innovators. Obviously, the output attributes are also strongly correlated in our data set (Table 3).

**Table 3:** Correlation coefficients between output attributes, significant correlations at *p*<0.05 are marked in italics.

	*y*_1_	*y*_2_	*y*_3_	*y*_4_	*y*_5_	*y*_6_
*y*_1_	1.000	*0*.*868*	*0*.*355*	*0*.*406*	*0*.*780*	*0*.*666*
*y*_2_	*0*.*868*	1.000	*0*.*383*	*0*.*477*	*0*.*648*	*0*.*423*
*y*_3_	*0*.*355*	*0*.*383*	1.000	*0*.*650*	*0*.*342*	0.088
*y*_4_	*0*.*406*	*0*.*477*	*0*.*650*	1.000	*0*.*688*	*0*.*167*
*y*_5_	*0*.*780*	*0*.*648*	*0*.*342*	*0*.*688*	1.000	*0*.*721*
*y*_6_	*0*.*666*	*0*.*423*	0.088	*0*.*167*	*0*.*721*	1.000

## Results

To avoid model overfitting, a set of 50 runs was generated using a holdout method of 70%, 15% and 15% of data used for training, validation, and testing, respectively. Several MLP architectures were tested, using the same set of 50 runs for each, thus avoiding problems associated with local minima. We followed a systematic approach, adding complexity to the network by increasing the number of neurons per layer and by increasing the number of hidden layers. This approach begins by selecting a small number of hidden neurons and then increased the number of hidden neurons until training and testing improved [66].

**Fig 3:**
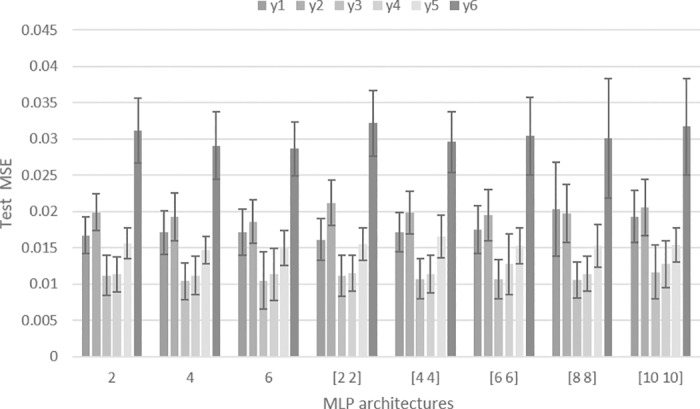
MSE for different multi-output MLP architectures. The average MSE and the standard deviation are presented for each output test datasets using 50 runs. MLP architectures with one single hidden layer are presented with a scalar value (representing the number of hidden neurons on that layer) while architectures with two hidden layers are presented by a vector of two values (the number of hidden neurons on the 1^st^ and 2^nd^ hidden layer).

The architecture that presented the lowest MSEs was the one that used only one hidden layer with six neurons (Fig 3). Analyzing the error per output, all structures presented a similar pattern: the smallest MSE was obtained for *y*_*3*_ and *y*_*4*_, while *y*_*2*_ and *y*_*6*_ presented higher estimation errors.

Further, we compared the performance of the multi-output MLP model with other models used in previous studies on innovation performance modeling (Table 4). Namely, we used single-output MLP [15], a three-stage least squares model (3SLS) [67], M5 regression tree [68], and adaptive neuro-fuzzy inference system (ANFIS) [16]. The single-output MLP models were trained using the same algorithm and setting as the multi-output one. In the M5 regression tree, the minimum number of instances to allow at a leaf node was set to four. Finally, the ANFIS was trained for various numbers of membership functions and if-then rules N = {2, 3, 5, 7}. The membership functions and if-then rules were generated automatically using a subtractive clustering algorithm. The adaptation of the premise and consequent parameters of the ANFIS was carried out by a gradient descent algorithm with one pass of the least-squares estimate.

**Table 4:** MSE (Mean ± SD) for each output–MIMO MLP vs. benchmark methods.

Output	MIMO MLP	MISO MLP	3SLS
*y*_*1*_	**0.01712 ± 0.00638**	0.01852 ± 0.00698	0.02289 ± 0.00356*
*y*_*2*_	**0.01858 ± 0.00599**	0.02182 ± 0.01080*	0.02663 ± 0.00331*
*y*_*3*_	**0.01048 ± 0.00783**	0.01076 ± 0.00607	0.01054 ± 0.00308
*y*_*4*_	**0.01137 ± 0.00718**	0.01383 ± 0.00994*	0.01172 ± 0.00288
*y*_*5*_	**0.01496 ± 0.00482**	0.01863 ± 0.00818*	0.01593 ± 0.00215
*y*_*6*_	0.02865 ± 0.00737	0.03793 ± 0.01212*	0.03092 ± 0.00309*
	M5 regression tree	ANFIS	
*y*_*1*_	0.01740 ± 0.00279	0.02264 ± 0.00371*	
*y*_*2*_	0.02196 ± 0.00551*	0.02932 ± 0.00359*	
*y*_*3*_	0.01210 ± 0.02578	0.12451 ± 0.00162*	
*y*_*4*_	0.01371 ± 0.02459*	0.01426 ± 0.00153*	
*y*_*5*_	0.01551 ± 0.00321	0.01910 ± 0.00293*	
*y*_*6*_	**0.02862 ± 0.00491**	0.03651 ± 0.00223*	

* significantly worse than MIMO MLP at *P* = 0.05 using Student’s paired samples *t*-test

To test the statistical differences between the best multi-output model and other models, we performed paired Student’s *t*-tests. The results indicated that the multi-output model with six neurons in the hidden layer performed significantly better at *p<0*.*05* than the single-output MLP model for outputs *y*_*2*_, *y*_*4*_, *y*_*5*_ and *y*_*6*_ (Table 4). Notably, the ANFIS was significantly outperformed for all six outputs. The multi-output model performed best for *y*_*1*_ to *y*_*5*_, whereas the M5 regression tree achieved the lowest MSE for *y*_*6*_.

An important issue in the design and implementation of a neural network is the sensitivity of its output to input and weight perturbations [69]. In this paper, we tested the MLP robustness by using three different set of input variables. The first set of variables used was the first six principal components, the second set included the first 12 principal components and in the third set the first 12 principal components and the six outputs for the year 2004 (*t-2*) was used.

Table 5 presents the average MSE for test data, per output using a 50 runs experiment for each set of input variables.

The results indicated that the set of 12 principal components and six outputs for *t*-2 performed better for all outputs except for *y*_*3*_ and *y*_*4*_ where just using the first 12 principal components performed slightly better.

**Table 5:** MSE (Mean ± SD) for each output–Different set of input variables.

Output	*CP*_1_-*CP*_6_	*CP*_1_-*CP*_12_	*CP*_1_-*CP*_12_ and *y*_1_^*t*-2^*-y*_6_^*t*-2^
*y*_*1*_	0.02033±0.00742	0.01984±0.00454	**0.01712±0.00638**
*y*_*2*_	0.02686±0.00978	0.02482±0.00624	**0.01858±0.00599**
*y*_*3*_	0.01082±0.00466	**0.00988±0.00417**	0.01048±0.00783
*y*_*4*_	0.01139±0.00522	**0.01119±0.00436**	0.01137±0.00718
*y*_*5*_	0.01875±0.008	0.01643±0.00396	**0.01496±0.00482**
*y*_*6*_	0.03598±0.00892	0.02987±0.0094	**0.02865±0.00737**

Sensitivity analysis is a method for extracting the cause and effect relationship between the inputs and outputs of the network. We started by varying each input variable (from its minimum to its maximum value), submitting the changed variable to the ANN while maintaining all other input variables at their mean values. Thus, we could assess the individual contributions of each variable.

We present a change indicator to summarize the results better. We calculated the change for each output while varying the input variable, using the following equation:

$$Change_{{y_{i}x_{j}}_{k}}=({y_{i}x_{j}}_{k+1}-{y_{i}x_{j}}_{k});\;i=\{1,2,3,4,5,6\};\;j=\{1,2,\ldots ,18\};k=\{1,2,3,4\},$$ (2)

where $Change_{{y_{i}x_{j}}_{k}}$ is the value of the change of the output *y*_*i*_ using the *k*^*th*^ value of variable *x*_*j*_.

**Fig 4:**
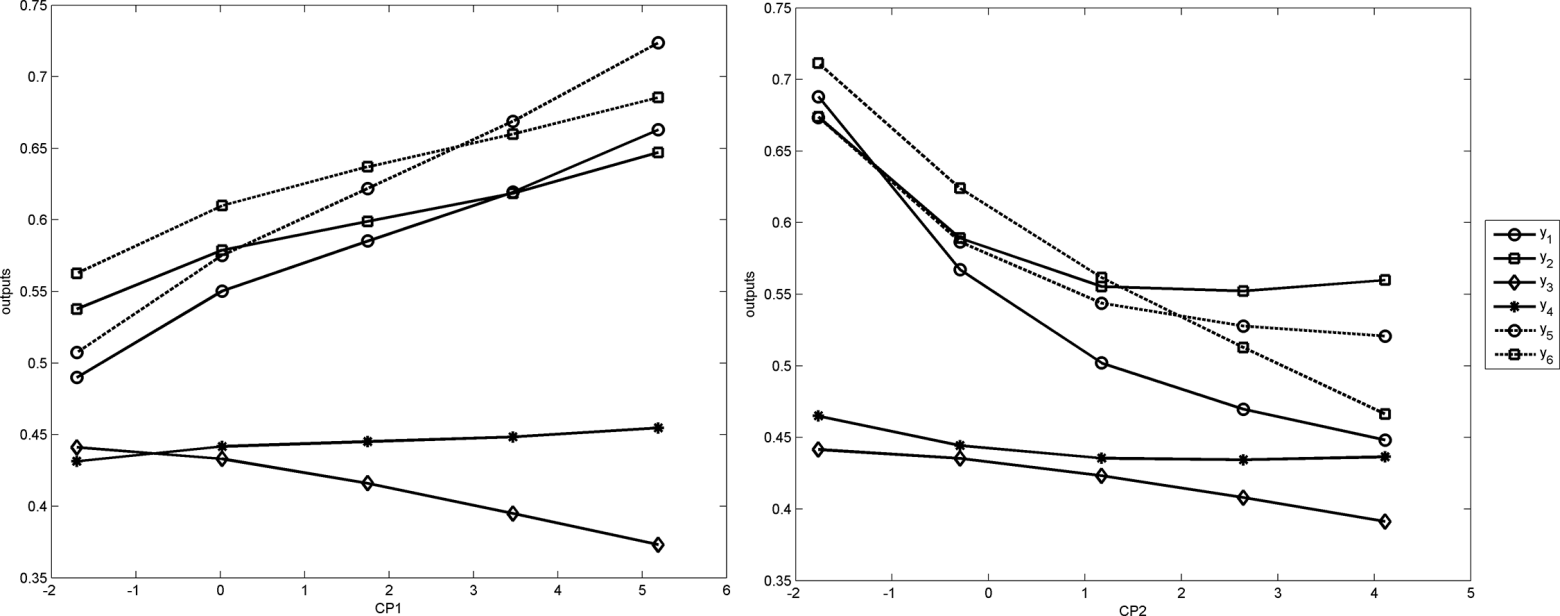
Sensitivity of multi-output ANN to the change of business R&D activity (*PC*_1_) and linkages (*PC*_2_). The tendency of the evolution was obtained by calculating the change for each output. Each variable *x*_*j*_ has five possible step values, meaning there are *k* = 4 possible changes of output values (i.e., from low to medium-low, from medium-low to medium, from medium to medium-high and from medium-high to high).

Fig 4 presents the tendency in the evolution of each output while varying one input variable at a time. The first two principal components are depicted in Fig 4, whereas the remaining components are presented in S3 Appendix.

The sensitivity analysis in S3 Appendix demonstrates that an increase in business R&D activity (*CP*_1_) has a positive effect on regional innovation performance. This effect is stronger when increasing from a low or medium-low value. A similar effect (sigmoidal curve) can also be observed for public R&D expenditures (*CP*_11_), which is, however, strongest when increased from a medium-low or medium value to a medium or medium-high value, respectively. The effect of linkages (*CP*_2_), on the other hand, was negative. Two different effects can be noted in the case of human resources, an inverted U-shape effect of secondary education (*CP*_3_), whereas the effect of tertiary education (*CP*_12_) was negative, particularly for low-performing regions. In other words, the effect of general skills was particularly large for regions with low levels of general skills. By contrast, when the region had a medium-high level of general skills available, further increases showed even a negative impact on regional innovation performance. Similarly, several diverse effects can be observed for inter-regional knowledge spillovers (*CP*_4_ and *CP*_7_). The effect was positive only for technological and non-technological inter-regional knowledge spillovers (*CP*_7_), and it worked only for the economic effects of innovation output (*y*_5_ and *y*_6_). Labour-market flexibility (*CP*_5_) promoted the outputs of innovation generators (*y*_1_ to *y*_4_) for low-performing regions in particular. The effect of economic diversification was positive only for the diffusion of new technologies (*y*_6_). FDI (*CP*_*8*_) promoted only resource-efficiency innovators (*y*_3_ and *y*_4_). Catching-up countries (*CP*_*9*_) showed a negative effect, suggesting that the New Member States did not exhibit strong convergence in regional innovativeness. Interestingly, the effect of entrepreneurship (*CP*_10_) was stronger for the outputs of innovation generators than for the economic effects of innovation activity. More specifically, the economic effects of regional innovation activity were promoted only in cases with a low level of entrepreneurship.

Altogether, significant effects were observed mainly for product and/or process innovators (*y*_1_) and marketing and/or organizational innovators (*y*_2_). The lowest effect of the extracted components was that of resource-efficiency innovators (*y*_3_ and *y*_4_). The observed effects were non-linear, providing support for the use of ANN in modeling innovation activity.

## Discussion and political implications

The research concerning forecasting innovation performance to date has tended to focus on firm-level rather than regional-level data. However, regional innovation performance is important for several reasons. The regional level is crucial for the implementation of innovation policies. Specifically, the European Regional Development Fund is the main instrument of European innovation and R&D policy, supporting regional innovation activity both directly, by co-financing R&D projects, and indirectly, by improving regional ICT and transport infrastructure. To make decisions about providing funding for innovation and R&D projects, it is increasingly important to consider their impact on regional innovation performance. Also, innovation activity is often carried out in regionally localized, collaborative networks. For policymakers, it is therefore important to identify highly innovative networks and to encourage regions to potentially transfer technology by improving the cooperation of networks.

The ready comprehensibility of the sensitivity analysis makes it possible to extract appropriate innovation strategies from the multi-output model. Thus, we develop the following important political implications. Our results suggest that specific innovation strategies must be developed based on the current level of input attributes in the region, which agrees with the priority of European innovation policy, namely regional smart specialization strategies. This finding also corroborates the observations of Tödtling and Trippl [6], suggesting that there is no single, ideal model for innovation policy in European regions.

This study provides empirical support that the business R&D activity has a sigmoidal effect (Fig 4). This effect was also observed by Yang *et al*. [70], suggesting that linear modeling methods fail to capture the full dynamics of the relationship between business R&D intensity and innovation activity. Interestingly, we demonstrate that this nonlinear relationship also holds for government R&D activity. In other words, the most effective R&D state support should be directed to regions with below-average and average R&D activity. One possible explanation for this phenomenon might be that highly innovative regions use innovation policy with an increased emphasis on sustainability (built on their experience with previous R&D support). On the other hand, supporting less innovative regions may lead to more radical efforts.

The cooperation in innovation efforts seems to be effective for highly cooperative regions only (Fig 4). Specifically, the results suggest that both the depth and breadth of the linkages is required to promote regional innovation performance. Therefore, it is important to facilitate strong networking activities among various innovation actors to achieve effective technology transfer. Our results also suggest that the support of inter-regional knowledge spillovers (technological and non-technological) may provide positive economic outcomes (see the effect of *CP*_4_ in S3 Appendix). Specifically, both new-to-firm and new-to-market innovators seem to make use of knowledge spillovers. The strongest effect was observed for regions that gain high knowledge spillovers from neighboring regions. Therefore, we argue for supporting cross-border partnerships in innovation activities, in particular between strong and weak RISs. For lagging behind regions, this may compensate for their weak innovation activity.

Furthermore, the development of training and life-long learning systems should be aimed especially at regions with a low level of general skills and/or labor-market flexibility (the inverted U-turn effects of *CP*_3_ and *CP*_5_ in S3 Appendix). An ineffective educational structure may also account for the lower effect of tertiary education in these regions (the negative effect of *CP*_12_ in S3 Appendix).

Another important finding was that an increase in entrepreneurship promotes generators of regional innovation, irrespective of regional innovativeness. The support of self-employment and business start-ups seems therefore to be an effective instrument of innovation policy, irrespective of the current level of entrepreneurial activity in the region (the effect of *CP*_10_ in S3 Appendix). The development of the entrepreneurial skills of students and researchers is therefore strongly recommended to promote the performance of regional innovators.

Overall, the present findings seem to be consistent with other research which found the firm’s technological competences (*CP*_1_) to be the main determinant of innovation activities [7,8]. Entrepreneurial activity (*CP*_10_) was another key determinant of regional innovation performance.

## Conclusions

Prior studies have noted the importance of various firm-related determinants for modeling innovation performance. However, such studies have been limited using single-output models. This study aimed to model the multi-output and nonlinear character of innovation performance. For this purpose, we developed a multi-output MLP-based model. It was hypothesized that this model could better explain the complex, non-linear patterns in regional innovation structures. The results of this study indicate that the multi-output MLP model better copes with this problem compared to single-output models such as MLP, 3SLS, M5 regression tree and ANFIS. Also, the current study found that the performance of the multi-output MLP model improves with the increasing number of the innovation performance determinants and the time-series components, respectively. Thus, the proposed nonlinear model of the innovation process can efficiently capture both the generation and the transfer of knowledge and technology at the firm and regional level.

The model also efficiently solves the problems of interdependencies among inputs and outputs. We showed that sensitivity analysis could be effectively employed to extract the knowledge stored in the multi-output MLP model. We believe that this study has gone some way towards enhancing our understanding of the role played by determinants in innovation performance modeling. Also, we believe this study provides important political implications and adds substantially to our understanding of regional innovation policy due to the extracted innovation strategies. These could be further used to develop targeted interventions aimed at improving innovation performance at the regional level. This is especially relevant for regions with a weak innovation performance that can profit from the knowledge spillovers from neighboring regions. The support of effective tertiary education and the development of entrepreneurial skills can also be recommended as appropriate policy measures. Most importantly, we observed a sigmoidal effect of government R&D activity, suggesting that the financial support of the European research and innovation policy should be focused on regions with below-average / average R&D activity. In other words, the support of weak performers, on one hand, and highly innovative regions, on the other hand, seems to be less effective.

Our findings in this study are subject to at least two limitations. First, the availability of regional-level data was limited. To obtain a consistent dataset, we had to use CIS 2004 and CIS 2006 data, because although CIS 2008 (and 2010) data were also available, the NUTS 2 membership of firms was missing from the most recent datasets. Second, the proposed model does and could not consider the relationships between the inputs. Therefore, we are currently in the process of investigating the potential of using recurrent ANNs for modeling regional innovation performance. Further research might also explore models of regional innovation performance in particular sectors, such as manufacturing or services. We also believe that the proposed multi-output ANN model can be generalized and provide guidelines to the development of intelligent systems in a wide range of related economic or sustainable development applications.

## Supporting information

S1 Appendix Preprocessed dataset. (DOCX)

S2 Appendix Component loadings. (DOCX)

S3 Appendix Sensitivity analysis of multi-output ANN. (DOCX)
