# Correction: Modelling innovation performance of European regions using multi-output neural networks

**DOI:** 10.1371/journal.pone.0189746

**Published:** 2017-12-08

**Authors:** Petr Hajek, Roberto Henriques

There is an error in affiliation 2 for author Roberto Henriques. Affiliation 2 should be: NOVA IMS, Universidade Nova de Lisboa, Lisboa, Portugal.
