# Supplementary material for: Modelling innovation performance of European regions using multi-output neural networks
Source: PLoS One. 2017 Oct 2;12(10):e0185755. doi: 10.1371/journal.pone.0185755 (PMC5624612; doi:10.1371/journal.pone.0185755)
Supplement: S1 Appendix — (DOCX) [file pone.0185755.s001.docx]

| S1 Appendix – Preprocessed dataset | | | | | | | | | | | | | | | | | | | | | | | | |
| --- | --- | --- | --- | --- | --- | --- | --- | --- | --- | --- | --- | --- | --- | --- | --- | --- | --- | --- | --- | --- | --- | --- | --- | --- |
|  | *CP*_1_ | *CP*_2_ | *CP*_3_ | *CP*_4_ | *CP*_5_ | *CP*_6_ | *CP*_7_ | *CP*_8_ | *CP*_9_ | *CP*_10_ | *CP*_11_ | *CP*_12_ | *y*_1_^2004^ | *y*_2_^2004^ | *y*_3_^2004^ | *y*_4_^2004^ | *y*_5_^2004^ | *y*_6_^2004^ | *y*_1_^2006^ | *y*_2_^2006^ | *y*_3_^2006^ | *y*_4_^2006^ | *y*_5_^2006^ | *y*_6_^2006^ |
| AT11 | -0.59 | -0.57 | 0.29 | -1.07 | 0.46 | -0.88 | -0.90 | -1.08 | -0.32 | -0.62 | 0.07 | -0.12 | -0.06 | -0.07 | -0.55 | -0.60 | -0.33 | -0.33 | 0.88 | 0.86 | 0.29 | 0.36 | 0.80 | 0.88 |
| AT12 | -0.09 | -0.40 | 0.43 | 0.04 | 0.76 | -1.05 | -0.51 | 0.10 | -0.74 | -0.45 | -0.19 | 0.10 | -0.02 | -0.07 | -0.55 | -0.48 | -0.33 | -0.24 | 0.88 | 0.86 | 0.29 | 0.36 | 0.80 | 0.88 |
| AT13 | 1.37 | 0.17 | 0.24 | -1.02 | 0.23 | 0.34 | -0.95 | -0.47 | -1.28 | 0.94 | 1.01 | 0.72 | -0.06 | -0.07 | -0.55 | -0.60 | -0.33 | -0.33 | 0.88 | 0.86 | 0.29 | 0.36 | 0.80 | 0.88 |
| AT21 | -0.13 | -0.37 | 0.45 | -0.26 | 0.88 | -1.23 | 0.02 | -0.72 | -0.56 | -0.70 | -0.11 | -0.54 | -0.02 | -0.07 | -0.68 | -0.48 | -0.35 | -0.24 | 0.84 | 0.89 | 0.36 | 0.32 | 0.70 | 0.74 |
| AT22 | 0.12 | -0.29 | 0.72 | -0.52 | 1.05 | -1.38 | 0.56 | -1.37 | 0.17 | 0.28 | 0.72 | -0.41 | -0.02 | -0.07 | -0.55 | -0.48 | -0.33 | -0.24 | 0.84 | 0.89 | 0.36 | 0.32 | 0.70 | 0.74 |
| AT31 | 0.02 | -0.69 | 0.93 | -0.40 | 1.31 | -1.61 | -0.33 | -0.41 | -0.72 | -0.26 | -0.77 | -0.62 | -0.02 | -0.07 | -0.55 | -0.48 | -0.33 | -0.24 | 0.89 | 0.84 | 0.36 | 0.44 | 0.83 | 0.92 |
| AT32 | -0.32 | -0.39 | 0.31 | -0.64 | 1.19 | -1.00 | -0.94 | 0.02 | -0.78 | -0.54 | -0.21 | 0.02 | -0.02 | -0.07 | -0.68 | -0.48 | -0.35 | -0.24 | 0.89 | 0.84 | 0.36 | 0.44 | 0.83 | 0.92 |
| AT33 | -0.27 | -0.63 | 0.41 | -1.07 | 1.15 | -1.28 | -1.12 | -0.73 | -0.29 | -0.18 | 0.04 | -0.10 | -0.02 | -0.07 | -0.68 | -0.48 | -0.35 | -0.28 | 0.89 | 0.84 | 0.36 | 0.44 | 0.83 | 0.92 |
| AT34 | 0.75 | 0.15 | 1.01 | -0.97 | 1.11 | -1.22 | -0.70 | -0.55 | -0.94 | -0.34 | -0.75 | -0.78 | -0.02 | -0.07 | -0.68 | -0.48 | -0.35 | -0.28 | 0.89 | 0.84 | 0.36 | 0.44 | 0.83 | 0.92 |
| BE10 | 0.74 | -0.26 | -1.28 | 1.17 | -0.81 | 3.33 | 1.33 | 1.68 | -1.52 | 0.56 | 0.41 | 1.12 | 0.00 | -0.08 | -0.39 | -0.42 | -0.28 | -0.31 | 0.75 | 0.79 | 0.47 | 0.49 | 0.49 | 0.35 |
| BE21 | 0.83 | -0.57 | 0.41 | -0.52 | 0.29 | 0.31 | -0.94 | -0.29 | -1.43 | 0.54 | -1.09 | 0.48 | 0.00 | -0.17 | -0.39 | -0.42 | -0.29 | -0.31 | 0.83 | 0.73 | 0.43 | 0.48 | 0.44 | 0.29 |
| BE22 | -0.32 | -0.67 | 0.73 | -0.59 | 0.33 | 0.88 | -0.80 | -0.81 | -0.86 | 0.16 | -0.86 | 0.23 | 0.00 | -0.17 | -0.36 | -0.42 | -0.29 | -0.31 | 0.83 | 0.73 | 0.43 | 0.48 | 0.44 | 0.29 |
| BE23 | 0.74 | -0.48 | 0.29 | -0.43 | 0.40 | 0.17 | -0.72 | -0.56 | -0.99 | 0.52 | -1.00 | 1.06 | 0.00 | -0.17 | -0.36 | -0.42 | -0.29 | -0.31 | 0.83 | 0.73 | 0.43 | 0.48 | 0.44 | 0.29 |
| BE24 | 0.88 | -0.49 | 0.35 | -0.55 | 0.63 | 0.53 | -0.05 | 0.09 | -1.29 | 0.52 | -0.65 | 1.25 | 0.00 | -0.17 | -0.36 | -0.42 | -0.28 | -0.31 | 0.83 | 0.73 | 0.43 | 0.48 | 0.44 | 0.29 |
| BE25 | -0.01 | -0.72 | 0.64 | -0.55 | 0.53 | 0.26 | -0.83 | -0.77 | -0.93 | 0.38 | -1.08 | 0.20 | 0.00 | -0.17 | -0.36 | -0.42 | -0.29 | -0.31 | 0.83 | 0.73 | 0.43 | 0.48 | 0.44 | 0.29 |
| BE31 | 2.71 | 0.49 | -1.40 | 0.20 | -1.20 | 2.01 | 2.27 | 0.76 | -1.13 | -0.10 | -0.70 | 3.49 | 0.00 | -0.17 | -0.36 | -0.42 | -0.29 | -0.31 | 0.82 | 0.70 | 0.49 | 0.52 | 1.00 | 0.42 |
| BE32 | 0.20 | -0.27 | -0.64 | -0.14 | -1.33 | 3.11 | 2.06 | -0.42 | -0.91 | -0.08 | -1.05 | 0.62 | 0.00 | -0.17 | -0.36 | -0.42 | -0.29 | -0.31 | 0.82 | 0.70 | 0.49 | 0.52 | 1.00 | 0.42 |
| BE33 | 0.46 | -0.14 | -0.88 | -0.10 | -1.16 | 2.72 | 2.08 | -0.30 | -0.88 | -0.20 | -0.90 | 0.97 | 0.00 | -0.17 | -0.36 | -0.42 | -0.29 | -0.31 | 0.82 | 0.70 | 0.49 | 0.52 | 1.00 | 0.42 |
| BE34 | -0.10 | -0.79 | -0.20 | -0.91 | -0.31 | 0.52 | -0.77 | -0.71 | -0.26 | -0.17 | -0.61 | 0.00 | -0.29 | -0.22 | -0.36 | -0.44 | -0.32 | -0.39 | 0.82 | 0.70 | 0.49 | 0.52 | 1.00 | 0.42 |
| BE35 | 0.17 | -0.58 | -0.12 | -0.77 | -1.04 | 2.45 | -0.77 | -0.85 | -0.08 | -0.04 | -0.87 | 0.58 | -0.29 | -0.22 | -0.36 | -0.44 | -0.32 | -0.39 | 0.82 | 0.70 | 0.49 | 0.52 | 1.00 | 0.42 |
| BG31 | 0.46 | 1.02 | 0.62 | 1.54 | -2.20 | 0.99 | -0.52 | 0.70 | 0.32 | -2.00 | -1.60 | 0.08 | -0.80 | -1.70 | -0.34 | -0.28 | -0.14 | -0.49 | 0.12 | 0.05 | 0.39 | 0.45 | 0.46 | 0.34 |
| BG32 | -0.08 | 0.82 | 0.75 | -0.61 | -1.89 | 0.25 | -1.14 | 0.67 | 0.22 | -0.95 | -1.07 | -0.20 | -1.40 | -1.70 | -0.38 | -0.30 | -0.35 | -0.64 | 0.12 | 0.05 | 0.39 | 0.45 | 0.46 | 0.34 |
| BG33 | -0.28 | 0.80 | -0.10 | -0.59 | -1.70 | -0.54 | -1.28 | 0.56 | 0.06 | -1.40 | -0.41 | -0.07 | -1.40 | -1.70 | -0.38 | -0.30 | -0.35 | -0.64 | 0.12 | 0.05 | 0.39 | 0.45 | 0.46 | 0.34 |
| BG34 | 0.41 | 0.90 | 0.21 | 1.49 | -1.78 | -0.10 | -0.52 | 0.88 | 0.23 | -1.97 | -1.57 | 0.24 | -0.80 | -1.70 | -0.34 | -0.28 | -0.14 | -0.49 | 0.12 | 0.05 | 0.39 | 0.45 | 0.46 | 0.34 |
| BG41 | -0.21 | 1.22 | 0.59 | -0.49 | -1.06 | -0.80 | -1.03 | 1.11 | -0.31 | -1.21 | 1.19 | 0.39 | -0.80 | -1.70 | -0.34 | -0.28 | -0.14 | -0.49 | 0.22 | 0.04 | 0.42 | 0.47 | 0.44 | 0.27 |
| BG42 | -0.32 | 0.79 | 0.76 | -0.58 | -1.54 | -0.31 | -0.86 | 0.54 | 0.04 | -0.47 | -1.00 | 0.02 | -0.80 | -1.70 | -0.34 | -0.28 | -0.14 | -0.49 | 0.22 | 0.04 | 0.42 | 0.47 | 0.44 | 0.27 |
| CY00 | 0.56 | 3.40 | -2.55 | 0.22 | 0.22 | -1.11 | -1.12 | 2.37 | -0.22 | -3.54 | -0.31 | 0.11 | -0.09 | -0.06 | -0.24 | -0.44 | -0.40 | -0.32 | 0.64 | 0.78 | 0.61 | 0.57 | 0.59 | 0.61 |
| CZ01 | -0.42 | 1.60 | 0.53 | 0.07 | 0.59 | -0.65 | -0.96 | 0.57 | -0.49 | -1.20 | 3.19 | 0.34 | -0.17 | -0.20 | -0.46 | -0.40 | -0.28 | -0.23 | 0.61 | 0.70 | 0.34 | 0.44 | 0.62 | 0.69 |
| CZ02 | -0.73 | 1.08 | 1.83 | 1.19 | 0.99 | -1.38 | 0.14 | -0.76 | -0.48 | -0.80 | 0.83 | -1.12 | -0.17 | -0.20 | -0.34 | -0.25 | -0.21 | -0.23 | 0.49 | 0.50 | 0.23 | 0.23 | 0.52 | 0.26 |
| CZ03 | -0.58 | 1.41 | 1.82 | 0.04 | 0.51 | -0.81 | 0.08 | -0.75 | -0.42 | -0.79 | -0.76 | -0.20 | -0.21 | -0.21 | -0.34 | -0.25 | -0.21 | -0.23 | 0.47 | 0.44 | 0.54 | 0.57 | 0.47 | 0.30 |
| CZ04 | -0.86 | 1.48 | 1.32 | 1.90 | 0.09 | -0.14 | 0.10 | -0.88 | -0.53 | -0.99 | 0.60 | -0.95 | -0.21 | -0.28 | -0.34 | -0.25 | -0.24 | -0.23 | 0.36 | 0.46 | 0.58 | 0.57 | 0.50 | 0.35 |
| CZ05 | -0.98 | 1.38 | 2.23 | 2.11 | 1.01 | -0.26 | 0.45 | -1.06 | -0.60 | -0.35 | -0.31 | -0.94 | -0.18 | -0.21 | -0.24 | -0.22 | -0.21 | -0.23 | 0.46 | 0.35 | 0.46 | 0.52 | 0.60 | 0.54 |
| CZ06 | -0.79 | 1.45 | 1.73 | 1.69 | 0.65 | -0.85 | -1.01 | -1.06 | -0.28 | -0.17 | 0.97 | -0.51 | -0.18 | -0.21 | -0.24 | -0.22 | -0.21 | -0.23 | 0.58 | 0.48 | 0.38 | 0.38 | 0.62 | 0.52 |
| CZ07 | -1.01 | 1.45 | 2.52 | -0.33 | 0.50 | -0.50 | -0.72 | -1.42 | -0.43 | 0.08 | -1.18 | 0.16 | -0.18 | -0.21 | -0.24 | -0.22 | -0.21 | -0.31 | 0.56 | 0.46 | 0.50 | 0.49 | 0.60 | 0.73 |
| CZ08 | -0.81 | 1.18 | 2.10 | 0.56 | 0.00 | 0.16 | -0.31 | -0.89 | -0.41 | -0.83 | 0.24 | -1.19 | -0.18 | -0.21 | -0.24 | -0.22 | -0.24 | -0.31 | 0.35 | 0.49 | 0.32 | 0.42 | 0.52 | 0.47 |
| DE11 | 3.63 | -0.53 | 1.40 | 0.16 | -0.23 | -0.85 | -0.92 | -0.26 | -0.65 | 0.14 | -1.50 | -1.74 | -0.19 | -0.21 | -0.24 | -0.20 | -0.25 | -0.29 | 0.98 | 0.89 | 0.47 | 0.64 | 0.99 | 0.82 |
| DE12 | 2.83 | -0.42 | 0.89 | -0.91 | -0.55 | -1.01 | 0.60 | -0.12 | -0.44 | -0.09 | 1.26 | -0.25 | -0.19 | -0.19 | -0.24 | -0.20 | -0.25 | -0.29 | 0.78 | 0.80 | 0.38 | 0.61 | 0.90 | 0.64 |
| DE13 | 1.86 | -0.59 | 0.89 | -0.58 | -0.26 | -0.53 | 0.24 | -0.31 | -0.51 | -0.38 | -0.31 | -0.52 | -0.23 | -0.24 | -0.24 | -0.20 | -0.25 | -0.29 | 0.85 | 0.85 | 0.47 | 0.63 | 0.89 | 0.77 |
| DE14 | 2.50 | -0.49 | 1.10 | -0.66 | -0.29 | -0.38 | 0.47 | -0.50 | -0.22 | -0.23 | -0.69 | -0.57 | -0.19 | -0.21 | -0.24 | -0.20 | -0.25 | -0.29 | 0.89 | 0.84 | 0.46 | 0.62 | 0.92 | 0.76 |
| DE21 | 4.02 | -0.44 | 0.29 | -0.18 | -0.51 | -1.30 | -0.98 | 0.32 | -0.29 | 0.60 | 0.21 | -0.44 | -0.21 | -0.18 | -0.25 | -0.25 | -0.28 | -0.32 | 0.96 | 1.00 | 0.46 | 0.65 | 1.00 | 0.74 |
| DE22 | -0.02 | -0.77 | 1.13 | -0.54 | 0.57 | -0.48 | 2.20 | -0.12 | -0.95 | -0.63 | -0.65 | -0.81 | -0.21 | -0.18 | -0.25 | -0.25 | -0.28 | -0.32 | 0.51 | 0.51 | 0.38 | 0.53 | 0.70 | 0.68 |
| DE23 | 2.25 | -0.68 | 1.00 | 0.29 | -0.33 | -0.59 | 0.39 | -0.34 | -0.10 | -0.76 | -0.88 | -0.71 | -0.21 | -0.18 | -0.25 | -0.25 | -0.28 | -0.32 | 0.85 | 0.80 | 0.49 | 0.61 | 0.90 | 0.87 |
| DE24 | 0.55 | -0.72 | 1.12 | 0.19 | -0.07 | -0.04 | 0.36 | -0.58 | -0.97 | -0.47 | -0.73 | -0.59 | -0.22 | -0.25 | -0.24 | -0.22 | -0.28 | -0.24 | 0.69 | 0.70 | 0.44 | 0.56 | 0.77 | 0.71 |
| DE25 | 2.24 | -0.60 | 0.63 | -0.23 | -0.36 | -0.75 | 0.38 | -0.20 | -0.57 | -0.27 | 0.01 | -0.68 | -0.19 | -0.21 | -0.24 | -0.22 | -0.28 | -0.32 | 0.92 | 0.98 | 0.49 | 0.65 | 0.95 | 0.78 |
| DE26 | 1.46 | -0.65 | 0.88 | -0.34 | 0.05 | -0.62 | 1.32 | -0.33 | -0.62 | -0.58 | -0.32 | -0.62 | -0.19 | -0.19 | -0.24 | -0.20 | -0.28 | -0.24 | 0.84 | 0.86 | 0.45 | 0.61 | 0.89 | 0.79 |
| DE27 | 0.47 | -0.74 | 0.97 | 0.22 | 0.47 | -0.65 | 1.60 | 0.08 | -0.84 | -0.49 | -0.18 | -0.82 | -0.19 | -0.18 | -0.24 | -0.21 | -0.28 | -0.29 | 0.84 | 0.88 | 0.51 | 0.62 | 0.87 | 0.83 |
| DE30 | 1.59 | -0.50 | 0.04 | -0.69 | -1.36 | 1.18 | -1.25 | -0.20 | -0.46 | 0.30 | 2.43 | 0.67 | -0.26 | -0.23 | -0.31 | -0.32 | -0.29 | -0.28 | 0.76 | 0.81 | 0.38 | 0.49 | 0.76 | 0.61 |
| DE41 | -0.57 | -0.81 | 0.01 | -0.63 | -0.63 | 0.07 | 1.94 | -0.02 | -0.42 | -1.45 | 2.31 | 0.17 | -0.26 | -0.23 | -0.29 | -0.30 | -0.29 | -0.26 | 0.50 | 0.55 | 0.41 | 0.50 | 0.62 | 0.54 |
| DE42 | -0.04 | -0.76 | -0.34 | -0.05 | -0.75 | -0.37 | 1.87 | -0.36 | -0.24 | -1.59 | 2.50 | 0.62 | -0.26 | -0.23 | -0.29 | -0.29 | -0.25 | -0.19 | 0.50 | 0.55 | 0.41 | 0.50 | 0.62 | 0.54 |
| DE50 | 0.46 | -0.61 | 0.84 | -0.59 | -0.79 | 1.66 | -0.90 | -0.66 | -2.18 | 0.53 | 0.54 | -0.06 | -0.27 | -0.27 | -0.29 | -0.28 | -0.25 | -0.31 | 0.62 | 0.75 | 0.33 | 0.46 | 0.69 | 0.57 |
| DE60 | 1.19 | -0.58 | 0.11 | 0.60 | -0.30 | 0.77 | -0.81 | 0.48 | -1.83 | 0.24 | 0.92 | 0.14 | -0.19 | -0.17 | -0.30 | -0.29 | -0.31 | -0.41 | 0.85 | 0.96 | 0.40 | 0.49 | 0.81 | 0.84 |
| DE71 | 2.26 | -0.56 | 0.53 | 1.83 | -0.14 | -0.06 | -1.03 | 0.25 | -1.26 | 0.48 | 0.26 | -0.53 | -0.19 | -0.19 | -0.24 | -0.20 | -0.25 | -0.29 | 0.98 | 1.00 | 0.46 | 0.58 | 0.98 | 0.86 |
| DE72 | 0.92 | -0.53 | 0.62 | -1.34 | -0.18 | 0.31 | 1.52 | -0.83 | -0.40 | -0.07 | 0.09 | 0.50 | -0.19 | -0.19 | -0.26 | -0.23 | -0.27 | -0.34 | 0.70 | 0.72 | 0.40 | 0.54 | 0.76 | 0.70 |
| DE73 | -0.18 | -0.73 | 0.76 | 0.74 | 0.05 | 0.61 | 2.09 | -0.39 | -0.76 | -0.72 | 0.09 | -0.36 | -0.19 | -0.19 | -0.26 | -0.23 | -0.23 | -0.24 | 0.51 | 0.46 | 0.33 | 0.45 | 0.63 | 0.63 |
| DE80 | -0.72 | -0.91 | -0.42 | -0.81 | -0.77 | -0.14 | 0.76 | -0.47 | 0.10 | -0.96 | 2.83 | 0.64 | -0.28 | -0.30 | -0.30 | -0.29 | -0.29 | -0.28 | 0.47 | 0.55 | 0.38 | 0.48 | 0.58 | 0.49 |
| DE91 | 1.76 | -0.55 | 1.60 | -1.03 | -0.96 | 0.24 | -0.92 | -1.10 | -0.08 | 0.20 | 1.01 | -0.04 | -0.23 | -0.22 | -0.28 | -0.23 | -0.23 | -0.24 | 0.71 | 0.74 | 0.31 | 0.49 | 0.77 | 0.51 |
| DE92 | 1.28 | -0.67 | 0.33 | 0.87 | -0.71 | 0.07 | -0.04 | -0.31 | -0.16 | -0.56 | 0.75 | 0.11 | -0.23 | -0.22 | -0.26 | -0.23 | -0.23 | -0.24 | 0.76 | 0.84 | 0.42 | 0.56 | 0.81 | 0.67 |
| DE93 | 0.13 | -0.80 | 0.22 | 1.04 | -0.07 | -0.33 | 1.80 | -0.10 | -0.57 | -1.21 | 0.31 | -0.13 | -0.19 | -0.17 | -0.28 | -0.23 | -0.23 | -0.24 | 0.66 | 0.73 | 0.41 | 0.52 | 0.75 | 0.71 |
| DE94 | -0.17 | -0.92 | 0.34 | 0.23 | -0.19 | 0.04 | 0.94 | -0.37 | -0.68 | -0.52 | -0.31 | -0.28 | -0.24 | -0.25 | -0.26 | -0.26 | -0.25 | -0.31 | 0.61 | 0.66 | 0.43 | 0.53 | 0.70 | 0.70 |
| DEA1 | 1.26 | -0.69 | 0.70 | 0.48 | -0.51 | 0.57 | -0.80 | -0.30 | -1.91 | 0.47 | -0.74 | -0.41 | -0.22 | -0.22 | -0.26 | -0.25 | -0.29 | -0.36 | 0.91 | 1.00 | 0.47 | 0.58 | 0.89 | 0.83 |
| DEA2 | 1.82 | -0.57 | 0.49 | -0.77 | -0.91 | 0.41 | -0.77 | -0.31 | -0.84 | 0.53 | 0.88 | -0.10 | -0.22 | -0.22 | -0.26 | -0.25 | -0.29 | -0.36 | 0.83 | 0.97 | 0.42 | 0.58 | 0.87 | 0.69 |
| DEA3 | -0.09 | -0.79 | 0.73 | -0.92 | -0.34 | 0.69 | 0.73 | -0.54 | -0.97 | -0.23 | 0.00 | -0.41 | -0.22 | -0.22 | -0.26 | -0.26 | -0.32 | -0.43 | 0.67 | 0.76 | 0.41 | 0.53 | 0.74 | 0.74 |
| DEA4 | 0.82 | -0.78 | 0.97 | -0.03 | -0.29 | 0.11 | -0.24 | -0.36 | -0.61 | -0.56 | -0.70 | -0.58 | -0.24 | -0.25 | -0.26 | -0.26 | -0.30 | -0.36 | 0.83 | 0.89 | 0.46 | 0.57 | 0.84 | 0.81 |
| DEA5 | 0.35 | -0.75 | 1.22 | -0.14 | -0.28 | 0.64 | 0.20 | -0.59 | -1.09 | -0.12 | -0.47 | -0.70 | -0.22 | -0.22 | -0.26 | -0.23 | -0.27 | -0.34 | 0.68 | 0.76 | 0.39 | 0.53 | 0.76 | 0.69 |
| DEB1 | -0.10 | -0.76 | 0.77 | 0.59 | 0.12 | 0.35 | 2.02 | -0.12 | -0.80 | -0.92 | -0.01 | -0.73 | -0.19 | -0.19 | -0.26 | -0.23 | -0.27 | -0.32 | 0.80 | 0.88 | 0.51 | 0.59 | 0.81 | 0.81 |
| DEB2 | -0.88 | -0.84 | 0.34 | -0.32 | 0.22 | 0.15 | 1.30 | -0.36 | -0.51 | -0.74 | 0.84 | -0.14 | -0.25 | -0.23 | -0.26 | -0.25 | -0.28 | -0.36 | 0.57 | 0.65 | 0.42 | 0.51 | 0.64 | 0.67 |
| DEB3 | 1.73 | -0.57 | 0.63 | -0.21 | -0.40 | 0.05 | 1.12 | -0.40 | -0.88 | -0.17 | -0.01 | -0.16 | -0.19 | -0.19 | -0.24 | -0.20 | -0.25 | -0.29 | 0.82 | 0.85 | 0.44 | 0.62 | 0.89 | 0.70 |
| DEC0 | 0.13 | -0.74 | 0.96 | 0.05 | -0.46 | 0.81 | -0.25 | -0.68 | -1.63 | -0.14 | 0.03 | -0.09 | -0.28 | -0.29 | -0.26 | -0.25 | -0.28 | -0.32 | 0.66 | 0.82 | 0.36 | 0.49 | 0.72 | 0.72 |
| DED1 | -0.46 | -0.88 | 0.82 | 0.38 | -0.40 | -0.50 | 0.76 | -0.77 | -0.56 | -1.04 | 2.16 | -0.25 | -0.27 | -0.30 | -0.28 | -0.25 | -0.25 | -0.19 | 0.43 | 0.38 | 0.37 | 0.45 | 0.57 | 0.46 |
| DED2 | 0.53 | -0.87 | 0.58 | -1.15 | -0.75 | -0.61 | -0.56 | -0.76 | 0.11 | -0.82 | 1.68 | -0.14 | -0.36 | -0.35 | -0.31 | -0.29 | -0.25 | -0.19 | 0.61 | 0.62 | 0.40 | 0.53 | 0.70 | 0.44 |
| DED3 | -0.51 | -0.86 | 0.18 | -0.33 | -0.62 | -0.14 | 0.15 | -0.60 | -0.26 | -0.87 | 2.62 | 0.35 | -0.36 | -0.35 | -0.31 | -0.29 | -0.25 | -0.19 | 0.31 | 0.34 | 0.30 | 0.44 | 0.53 | 0.37 |
| DEE0 | -0.86 | -0.72 | -0.13 | 0.11 | -0.75 | 0.20 | 2.84 | -0.62 | -0.07 | -1.02 | 2.81 | 0.46 | -0.23 | -0.22 | -0.28 | -0.23 | -0.23 | -0.23 | 0.49 | 0.57 | 0.43 | 0.50 | 0.58 | 0.48 |
| DEF0 | 0.02 | -0.78 | -0.04 | -0.47 | -0.13 | 0.00 | 1.45 | -0.06 | -0.70 | -0.62 | 0.56 | -0.06 | -0.19 | -0.17 | -0.30 | -0.29 | -0.29 | -0.28 | 0.66 | 0.75 | 0.41 | 0.53 | 0.72 | 0.69 |
| DEG0 | -0.13 | -0.69 | 0.36 | 0.01 | -0.45 | -0.24 | 2.07 | -0.48 | -0.11 | -0.95 | 2.24 | 0.16 | -0.23 | -0.22 | -0.24 | -0.22 | -0.23 | -0.23 | 0.55 | 0.51 | 0.42 | 0.54 | 0.66 | 0.52 |
| DK01 | 3.77 | 1.98 | -0.49 | -0.42 | 0.77 | -0.05 | -0.28 | -0.98 | -0.28 | 0.51 | -0.12 | 5.92 | -0.09 | 0.00 | -0.43 | -0.49 | -0.28 | -0.36 | 0.59 | 0.68 | 0.32 | 0.35 | 0.40 | 0.20 |
| DK02 | -0.94 | 0.16 | 0.67 | -0.64 | 1.84 | -0.17 | -0.57 | -0.87 | -0.59 | -0.57 | -0.31 | 5.56 | -0.09 | 0.00 | -0.43 | -0.49 | -0.28 | -0.36 | 0.59 | 0.68 | 0.32 | 0.35 | 0.40 | 0.20 |
| DK03 | -0.99 | 0.01 | 0.67 | -0.63 | 1.83 | 0.14 | -0.57 | -0.58 | -0.58 | -0.30 | -0.77 | 3.65 | -0.09 | 0.00 | -0.43 | -0.49 | -0.28 | -0.36 | 0.59 | 0.68 | 0.32 | 0.35 | 0.40 | 0.20 |
| DK04 | 0.02 | 0.56 | 0.45 | -0.80 | 1.65 | 0.04 | -0.50 | -0.96 | -0.09 | 0.00 | -0.38 | 4.14 | -0.09 | 0.00 | -0.43 | -0.49 | -0.28 | -0.36 | 0.59 | 0.68 | 0.32 | 0.35 | 0.40 | 0.20 |
| DK05 | -1.09 | -0.23 | 0.55 | -0.71 | 1.76 | -0.70 | -0.67 | -1.15 | -0.42 | -0.82 | 0.08 | 7.68 | -0.09 | 0.00 | -0.43 | -0.49 | -0.28 | -0.36 | 0.59 | 0.68 | 0.32 | 0.35 | 0.40 | 0.20 |
| EE00 | -0.48 | -0.17 | 0.98 | -0.56 | -0.10 | -0.89 | -1.17 | 0.03 | -0.29 | -1.13 | 0.22 | -0.01 | -0.08 | -0.15 | -0.40 | -0.36 | -0.24 | -0.19 | 0.83 | 0.74 | 0.37 | 0.34 | 0.54 | 0.80 |
| ES11 | 0.34 | -0.56 | -2.05 | 1.30 | -0.64 | -1.39 | 0.94 | -0.10 | 0.04 | -0.11 | -0.86 | 0.55 | -0.27 | -0.41 | -0.42 | -0.46 | -0.30 | -0.11 | 0.33 | 0.29 | 0.48 | 0.51 | 0.43 | 0.71 |
| ES12 | -0.19 | -0.49 | -1.63 | 1.29 | -0.59 | -0.72 | -0.25 | -0.57 | -0.36 | -0.82 | -0.26 | 0.34 | -0.27 | -0.41 | -0.42 | -0.46 | -0.30 | -0.11 | 0.41 | 0.42 | 0.33 | 0.38 | 0.58 | 0.72 |
| ES13 | 0.03 | -0.47 | -1.64 | 3.44 | -0.26 | -0.93 | 0.55 | -0.28 | -0.52 | -1.71 | -0.41 | 0.57 | -0.20 | -0.41 | -0.33 | -0.34 | -0.30 | -0.11 | 0.49 | 0.32 | 0.38 | 0.39 | 0.48 | 0.47 |
| ES21 | 0.59 | -0.22 | -0.70 | 0.71 | 0.11 | -0.46 | -0.38 | -0.05 | -1.18 | -0.34 | -1.26 | 0.17 | -0.20 | -0.36 | -0.33 | -0.34 | -0.28 | -0.11 | 0.53 | 0.38 | 0.51 | 0.41 | 0.49 | 0.68 |
| ES22 | 0.40 | -0.38 | -0.77 | 0.24 | 0.25 | -0.95 | 0.27 | -0.26 | -0.33 | -0.10 | -0.83 | 0.29 | -0.20 | -0.35 | -0.31 | -0.34 | -0.28 | -0.17 | 0.63 | 0.42 | 0.40 | 0.42 | 0.54 | 0.72 |
| ES23 | -0.45 | -0.39 | -1.20 | 2.36 | 0.40 | -1.22 | 2.31 | 0.04 | -0.46 | -1.12 | 0.14 | 0.17 | -0.20 | -0.35 | -0.31 | -0.34 | -0.28 | -0.11 | 0.49 | 0.38 | 0.38 | 0.42 | 0.55 | 0.50 |
| ES24 | -0.09 | -0.54 | -1.15 | 1.37 | 0.08 | -1.04 | -0.42 | -0.14 | -0.19 | -0.44 | -0.33 | -0.14 | -0.24 | -0.35 | -0.31 | -0.27 | -0.28 | -0.11 | 0.50 | 0.40 | 0.38 | 0.38 | 0.48 | 0.63 |
| ES30 | 0.77 | -0.20 | -1.69 | 3.50 | 0.24 | -0.34 | -1.50 | 0.17 | -1.52 | 0.14 | 0.11 | 0.01 | -0.24 | -0.30 | -0.39 | -0.27 | -0.37 | -0.11 | 0.41 | 0.47 | 0.32 | 0.38 | 0.43 | 0.59 |
| ES41 | -0.86 | -0.50 | -1.30 | 0.82 | 0.19 | -1.27 | 1.63 | -0.58 | 0.24 | 0.51 | 0.87 | 0.26 | -0.20 | -0.30 | -0.33 | -0.27 | -0.28 | -0.11 | 0.40 | 0.35 | 0.35 | 0.37 | 0.43 | 0.65 |
| ES42 | -0.62 | -0.40 | -2.18 | 0.55 | 0.06 | -1.79 | 3.33 | -0.17 | 0.25 | -0.59 | 0.33 | 0.25 | -0.16 | -0.30 | -0.31 | -0.27 | -0.34 | -0.11 | 0.37 | 0.29 | 0.40 | 0.36 | 0.41 | 0.62 |
| ES43 | -0.82 | -0.73 | -2.36 | 1.96 | -0.67 | -0.93 | 0.29 | -0.51 | 0.78 | -0.53 | 0.83 | -0.04 | -0.35 | -0.41 | -0.39 | -0.27 | -0.37 | -0.11 | 0.25 | 0.34 | 0.36 | 0.42 | 0.33 | 0.47 |
| ES51 | 0.34 | -0.38 | -1.30 | 0.91 | 0.39 | -1.34 | -0.78 | 0.35 | -0.94 | 1.15 | -0.45 | -0.89 | -0.24 | -0.35 | -0.31 | -0.47 | -0.33 | -0.11 | 0.58 | 0.49 | 0.32 | 0.36 | 0.53 | 0.62 |
| ES52 | -0.97 | -0.28 | -1.61 | 1.02 | 0.76 | -1.35 | 1.20 | -0.32 | -0.45 | 0.91 | 1.26 | -0.41 | -0.16 | -0.32 | -0.31 | -0.27 | -0.33 | -0.11 | 0.46 | 0.41 | 0.42 | 0.39 | 0.56 | 0.65 |
| ES53 | -1.15 | -0.40 | -2.21 | -0.39 | 0.41 | -0.85 | -1.20 | -0.25 | -0.56 | -1.19 | -0.62 | -0.37 | -0.34 | -0.48 | -0.68 | -0.70 | -0.57 | -1.15 | 0.23 | 0.26 | 0.31 | 0.42 | 0.69 | 0.78 |
| ES61 | -0.75 | -0.42 | -2.59 | 1.13 | -0.16 | -1.06 | 1.24 | -0.30 | 0.07 | 1.17 | 0.77 | 0.06 | -0.16 | -0.32 | -0.39 | -0.27 | -0.37 | -0.23 | 0.38 | 0.38 | 0.33 | 0.40 | 0.45 | 0.58 |
| ES62 | -0.88 | -0.77 | -1.23 | 1.95 | 0.65 | -1.86 | -1.29 | -1.13 | -0.08 | 0.36 | -0.36 | 0.24 | -0.16 | -0.32 | -0.39 | -0.27 | -0.34 | -0.11 | 0.33 | 0.35 | 0.42 | 0.37 | 0.36 | 0.43 |
| ES63 | -0.96 | -0.16 | -1.08 | 6.20 | -0.28 | 4.52 | -1.53 | -1.15 | 0.15 | -0.93 | -2.03 | -0.21 | -0.34 | -0.64 | -0.48 | -0.49 | -0.48 | -0.24 | 0.72 | 0.35 | 0.25 | 0.00 | 0.13 | 1.00 |
| ES64 | -1.02 | -0.10 | -0.91 | -0.22 | -1.78 | 5.64 | -1.14 | -0.29 | 0.05 | -0.63 | -1.50 | -0.78 | -0.55 | -0.82 | -0.53 | -0.53 | -0.35 | -0.50 | 0.08 | 0.03 | 0.25 | 0.21 | 0.20 | 0.55 |
| ES70 | -0.86 | -0.46 | -2.59 | -0.44 | -0.10 | -0.64 | -1.19 | -0.33 | -0.46 | -1.26 | 0.01 | -0.27 | -0.40 | -0.60 | -0.15 | -0.14 | -0.62 | -0.96 | 0.26 | 0.35 | 0.44 | 0.37 | 0.26 | 0.32 |
| FI13 | -0.22 | -0.45 | 0.06 | -0.57 | 0.81 | 0.77 | 0.18 | -0.73 | 3.62 | 0.13 | 1.01 | -0.03 | -0.17 | -0.10 | -0.42 | -0.42 | -0.20 | -0.30 | 0.70 | 0.56 | 0.39 | 0.33 | 0.65 | 0.76 |
| FI18 | 3.21 | 0.06 | -0.68 | 1.28 | 0.79 | -0.34 | -0.89 | 0.08 | 3.03 | -0.50 | 0.40 | -0.06 | -0.17 | -0.10 | -0.42 | -0.42 | -0.20 | -0.30 | 0.82 | 0.59 | 0.33 | 0.28 | 0.82 | 0.88 |
| FI19 | 1.80 | -0.42 | 0.23 | -0.18 | 1.02 | -0.24 | 0.01 | -0.52 | 3.50 | -0.53 | -0.23 | -0.94 | -0.17 | -0.10 | -0.42 | -0.42 | -0.20 | -0.30 | 0.83 | 0.55 | 0.31 | 0.31 | 0.79 | 0.95 |
| FI1A | 2.71 | -0.15 | -0.77 | 1.44 | 0.37 | 0.29 | 0.95 | -0.47 | 4.22 | -1.12 | 0.24 | -0.36 | -0.17 | -0.10 | -0.42 | -0.42 | -0.20 | -0.30 | 0.79 | 0.63 | 0.39 | 0.35 | 0.73 | 0.80 |
| FR10 | 2.01 | -0.21 | -0.81 | -0.46 | -0.85 | 0.84 | -0.78 | 1.38 | -0.41 | 1.38 | 1.53 | -0.55 | -0.54 | -0.22 | -0.19 | -0.28 | -0.34 | -0.59 | 0.97 | 1.00 | 0.51 | 0.59 | 0.93 | 0.76 |
| FR21 | -0.37 | -0.88 | -0.23 | -1.14 | -0.65 | 0.97 | 2.12 | 0.59 | 0.18 | -0.80 | -0.26 | -0.84 | -0.41 | -0.21 | -0.19 | -0.28 | -0.34 | -0.59 | 0.72 | 0.76 | 0.46 | 0.51 | 0.71 | 0.70 |
| FR22 | -0.02 | -1.02 | 0.10 | -0.82 | -0.51 | 0.51 | 1.38 | 0.44 | -0.18 | -0.88 | -0.67 | -1.38 | -0.54 | -0.22 | -0.19 | -0.25 | -0.34 | -0.54 | 0.72 | 0.76 | 0.46 | 0.51 | 0.71 | 0.70 |
| FR23 | 0.19 | -0.39 | 0.24 | -1.25 | -0.43 | 0.84 | 1.65 | 0.23 | -0.20 | -0.78 | -0.35 | -1.46 | -0.54 | -0.22 | -0.19 | -0.28 | -0.34 | -0.59 | 0.72 | 0.76 | 0.46 | 0.51 | 0.71 | 0.70 |
| FR24 | 0.36 | -0.69 | -0.39 | -0.75 | -0.54 | 0.43 | 2.15 | 0.43 | 0.16 | -0.96 | -0.28 | -0.87 | -0.38 | -0.22 | -0.19 | -0.28 | -0.31 | -0.48 | 0.72 | 0.76 | 0.46 | 0.51 | 0.71 | 0.70 |
| FR25 | 0.26 | -0.67 | -0.57 | -0.83 | -1.05 | 0.60 | 0.35 | 0.24 | 0.49 | -0.73 | -0.32 | -0.70 | -0.49 | -0.28 | -0.20 | -0.28 | -0.36 | -0.49 | 0.72 | 0.76 | 0.46 | 0.51 | 0.71 | 0.70 |
| FR26 | -0.04 | -0.92 | -0.34 | -0.90 | -0.55 | 0.17 | 2.21 | 0.44 | 0.14 | -1.03 | -0.01 | -0.78 | -0.41 | -0.21 | -0.19 | -0.28 | -0.34 | -0.48 | 0.72 | 0.76 | 0.46 | 0.51 | 0.71 | 0.70 |
| FR30 | -0.11 | -0.69 | -0.16 | -0.77 | -1.12 | 1.32 | -0.52 | 0.34 | -0.19 | -0.45 | -0.09 | -0.78 | -0.55 | -0.28 | -0.19 | -0.25 | -0.38 | -0.54 | 0.54 | 0.61 | 0.42 | 0.47 | 0.60 | 0.62 |
| FR41 | -0.34 | -0.41 | 0.21 | -1.11 | -0.79 | 1.15 | -0.71 | -0.47 | 0.12 | -0.04 | -0.17 | -0.69 | -0.41 | -0.21 | -0.19 | -0.28 | -0.35 | -0.59 | 0.58 | 0.58 | 0.40 | 0.51 | 0.67 | 0.60 |
| FR42 | 0.26 | -0.10 | 0.27 | -1.22 | -0.19 | 0.32 | -0.61 | -0.18 | -0.13 | -0.13 | -0.03 | -0.38 | -0.41 | -0.21 | -0.19 | -0.30 | -0.35 | -0.59 | 0.58 | 0.58 | 0.40 | 0.51 | 0.67 | 0.60 |
| FR43 | 0.21 | -0.36 | 0.76 | -0.77 | -0.50 | 1.01 | -0.42 | -0.33 | 0.10 | -0.20 | -1.04 | -1.09 | -0.41 | -0.21 | -0.19 | -0.28 | -0.35 | -0.48 | 0.58 | 0.58 | 0.40 | 0.51 | 0.67 | 0.60 |
| FR51 | -0.31 | -0.63 | 0.02 | -0.71 | -0.34 | 0.24 | -0.69 | 0.04 | 0.01 | 0.19 | 0.03 | -0.48 | -0.49 | -0.28 | -0.20 | -0.28 | -0.36 | -0.49 | 0.64 | 0.65 | 0.46 | 0.51 | 0.67 | 0.67 |
| FR52 | 0.32 | -1.21 | 0.03 | -0.59 | -0.55 | -0.38 | -0.93 | -0.03 | 0.65 | 0.04 | 0.17 | -0.52 | -0.49 | -0.28 | -0.20 | -0.28 | -0.36 | -0.49 | 0.64 | 0.65 | 0.46 | 0.51 | 0.67 | 0.67 |
| FR53 | -0.34 | -0.87 | -0.19 | -0.47 | -0.69 | 0.41 | -0.05 | -0.02 | 0.28 | -0.23 | -0.09 | -0.09 | -0.38 | -0.24 | -0.20 | -0.28 | -0.31 | -0.49 | 0.64 | 0.65 | 0.46 | 0.51 | 0.67 | 0.67 |
| FR61 | -0.46 | -0.75 | 0.05 | 0.29 | -0.68 | 1.31 | -0.74 | -0.22 | 0.38 | 0.51 | 0.36 | -0.48 | -0.38 | -0.24 | -0.21 | -0.34 | -0.31 | -0.49 | 0.79 | 0.84 | 0.48 | 0.53 | 0.73 | 0.63 |
| FR62 | 0.66 | -0.55 | 0.13 | 0.48 | -0.86 | 0.01 | -0.96 | -0.23 | 0.82 | 0.50 | 2.56 | -0.35 | -0.38 | -0.24 | -0.21 | -0.28 | -0.31 | -0.48 | 0.79 | 0.84 | 0.48 | 0.53 | 0.73 | 0.63 |
| FR63 | -0.88 | -0.70 | 0.24 | 0.62 | -0.50 | 1.00 | -0.65 | -0.51 | 0.43 | 0.01 | 0.38 | -0.44 | -0.38 | -0.24 | -0.20 | -0.28 | -0.31 | -0.48 | 0.79 | 0.84 | 0.48 | 0.53 | 0.73 | 0.63 |
| FR71 | 1.09 | -0.04 | -0.30 | -1.02 | -0.67 | 0.21 | 0.40 | 0.17 | 0.13 | 0.45 | 0.72 | -0.51 | -0.41 | -0.21 | -0.19 | -0.28 | -0.35 | -0.48 | 0.84 | 0.87 | 0.50 | 0.59 | 0.82 | 0.70 |
| FR72 | 0.50 | -0.80 | 0.07 | -0.80 | -0.87 | 0.17 | 0.09 | 0.16 | 0.36 | -0.56 | -0.37 | -0.42 | -0.38 | -0.24 | -0.20 | -0.28 | -0.31 | -0.48 | 0.84 | 0.87 | 0.50 | 0.59 | 0.82 | 0.70 |
| FR81 | -0.07 | -0.81 | -0.97 | 0.01 | -1.83 | 1.92 | 0.11 | 0.02 | 0.73 | -0.40 | 2.12 | 0.24 | -0.38 | -0.24 | -0.21 | -0.28 | -0.31 | -0.48 | 0.74 | 0.85 | 0.47 | 0.53 | 0.72 | 0.62 |
| FR82 | 0.23 | -1.01 | -0.68 | 0.10 | -1.13 | 1.05 | -0.65 | 0.42 | 0.15 | 0.00 | 1.30 | -0.57 | -0.57 | -0.24 | -0.21 | -0.28 | -0.35 | -0.48 | 0.74 | 0.85 | 0.47 | 0.53 | 0.72 | 0.62 |
| GR11 | -0.12 | -0.43 | -0.92 | -0.68 | -1.87 | -0.95 | -0.38 | 0.17 | 1.11 | 0.65 | -0.60 | 0.42 | -0.63 | -0.54 | -0.39 | -0.44 | -0.35 | -0.28 | 0.49 | 0.63 | 0.38 | 0.62 | 0.83 | 0.97 |
| GR12 | -0.20 | 0.19 | -0.83 | -1.01 | -1.28 | -0.92 | -1.19 | -0.20 | 0.24 | 0.83 | -0.13 | -0.34 | -0.63 | -0.54 | -0.39 | -0.44 | -0.35 | -0.28 | 0.58 | 0.74 | 0.63 | 0.59 | 0.59 | 0.21 |
| GR13 | -0.02 | -0.05 | -1.00 | -0.59 | -1.78 | -1.25 | 0.05 | -0.46 | 0.59 | -0.24 | -0.54 | -0.08 | -0.63 | -0.50 | -0.39 | -0.44 | -0.35 | -0.28 | 0.49 | 0.63 | 0.38 | 0.62 | 0.83 | 0.97 |
| GR14 | -0.17 | -0.29 | -1.25 | -0.65 | -1.81 | -1.10 | -0.22 | 0.06 | 0.87 | 0.36 | -0.38 | 0.43 | -0.63 | -0.50 | -0.39 | -0.44 | -0.35 | -0.28 | 0.49 | 0.63 | 0.38 | 0.62 | 0.83 | 0.97 |
| GR21 | -0.43 | -0.02 | -1.33 | -1.14 | -1.77 | -0.63 | -1.09 | -0.85 | 0.83 | 0.46 | 0.02 | 0.25 | -0.64 | -0.50 | -0.39 | -0.44 | -0.40 | -0.35 | 0.60 | 0.99 | 0.37 | 0.20 | 0.37 | 0.68 |
| GR22 | -1.07 | -0.13 | -2.14 | -0.80 | -0.96 | -0.93 | -1.63 | -0.26 | 0.00 | -0.60 | -0.69 | -0.37 | -0.64 | -0.50 | -0.41 | -0.48 | -0.40 | -0.35 | 0.60 | 0.99 | 0.37 | 0.20 | 0.37 | 0.68 |
| GR23 | -0.45 | -0.40 | -1.27 | -1.42 | -1.89 | -1.11 | -1.23 | -0.61 | 1.11 | 0.81 | 0.05 | 0.42 | -0.64 | -0.50 | -0.41 | -0.48 | -0.40 | -0.35 | 0.60 | 0.99 | 0.37 | 0.20 | 0.37 | 0.68 |
| GR24 | -0.38 | -0.20 | -0.97 | -0.78 | -1.29 | -1.80 | 0.41 | -0.26 | 0.37 | 0.00 | -0.37 | -0.20 | -0.52 | -0.46 | -0.39 | -0.44 | -0.35 | -0.30 | 0.60 | 0.99 | 0.37 | 0.20 | 0.37 | 0.68 |
| GR25 | -0.43 | -0.63 | -1.02 | -0.98 | -1.70 | -1.39 | 0.32 | 0.27 | 0.99 | 0.78 | -0.44 | 0.49 | -0.52 | -0.46 | -0.41 | -0.44 | -0.35 | -0.30 | 0.60 | 0.99 | 0.37 | 0.20 | 0.37 | 0.68 |
| GR30 | -0.15 | 1.03 | -0.91 | -0.22 | -0.65 | 0.25 | -1.30 | -0.15 | -0.95 | 0.74 | 0.54 | -1.06 | -0.52 | -0.46 | -0.41 | -0.44 | -0.35 | -0.30 | 0.67 | 0.80 | 0.56 | 0.54 | 0.77 | 0.47 |
| GR41 | -0.57 | 0.25 | -1.39 | -1.03 | -1.85 | 0.50 | -1.35 | -0.55 | 0.55 | -0.09 | -0.39 | -0.17 | -0.65 | -0.48 | -0.38 | -0.43 | -0.40 | -0.33 | 0.82 | 0.95 | 1.00 | 1.00 | 0.65 | 0.15 |
| GR42 | -0.95 | 0.36 | -2.05 | -0.70 | -0.68 | -0.63 | -1.49 | -0.40 | -0.38 | -1.09 | -0.64 | -0.83 | -0.65 | -0.48 | -0.38 | -0.43 | -0.40 | -0.33 | 0.82 | 0.95 | 1.00 | 1.00 | 0.65 | 0.15 |
| GR43 | -0.47 | -0.30 | -1.29 | -1.24 | -1.08 | -1.61 | -1.30 | 0.10 | 0.75 | 0.41 | 0.38 | 0.38 | -0.65 | -0.48 | -0.38 | -0.43 | -0.40 | -0.33 | 0.82 | 0.95 | 1.00 | 1.00 | 0.65 | 0.15 |
| HU10 | -0.09 | 1.97 | 0.08 | 1.03 | -0.22 | -0.12 | -0.79 | 1.00 | -0.33 | -0.69 | 1.87 | 0.16 | -0.32 | -0.34 | -0.37 | -0.32 | -0.22 | -0.30 | 0.23 | 0.39 | 0.20 | 0.32 | 0.42 | 0.43 |
| HU21 | -0.72 | 1.83 | 1.32 | -0.48 | 0.36 | -0.26 | 1.40 | -0.16 | -0.52 | -0.44 | -0.86 | -0.37 | -0.32 | -0.34 | -0.37 | -0.32 | -0.22 | -0.30 | 0.23 | 0.29 | 0.27 | 0.43 | 0.45 | 0.30 |
| HU22 | -0.42 | 1.76 | 1.07 | -0.19 | -0.16 | -0.15 | -0.14 | 0.05 | -0.33 | -0.94 | -1.11 | -0.21 | -0.43 | -0.49 | -0.42 | -0.32 | -0.22 | -0.30 | 0.10 | 0.27 | 0.24 | 0.29 | 0.29 | 0.43 |
| HU23 | -0.51 | 1.54 | 0.47 | 0.48 | -0.83 | -0.06 | -0.05 | 0.06 | 0.54 | -0.85 | 0.44 | 0.39 | -0.43 | -0.42 | -0.37 | -0.32 | -0.22 | -0.30 | 0.09 | 0.21 | 0.14 | 0.31 | 0.44 | 0.48 |
| HU31 | -0.68 | 1.81 | 0.95 | -0.54 | -0.41 | 0.45 | 1.17 | -0.43 | -0.40 | -0.55 | -0.83 | -0.43 | -0.32 | -0.34 | -0.39 | -0.35 | -0.26 | -0.33 | 0.05 | 0.30 | 0.24 | 0.37 | 0.37 | 0.32 |
| HU32 | -0.47 | 1.61 | 0.44 | -0.76 | -0.61 | -0.17 | 1.09 | -0.15 | 0.41 | -0.62 | -0.20 | 0.00 | -0.32 | -0.34 | -0.39 | -0.35 | -0.26 | -0.33 | 0.07 | 0.16 | 0.16 | 0.26 | 0.35 | 0.29 |
| HU33 | -0.35 | 1.53 | 0.20 | 0.19 | -0.91 | -0.57 | 1.66 | 0.42 | 0.77 | -1.02 | 0.98 | 0.65 | -0.32 | -0.34 | -0.37 | -0.32 | -0.22 | -0.30 | 0.14 | 0.26 | 0.21 | 0.29 | 0.43 | 0.26 |
| IE01 | -0.28 | -1.14 | -0.81 | -1.13 | -0.10 | -1.80 | 0.06 | -0.20 | 0.36 | -0.40 | -0.08 | -0.24 | -0.37 | -0.35 | -0.35 | -0.37 | -0.30 | -0.28 | 0.65 | 0.64 | 0.52 | 0.55 | 0.68 | 0.57 |
| IE02 | -0.38 | -1.05 | -0.72 | -0.84 | 0.47 | -1.37 | -0.53 | 1.17 | -0.34 | 0.67 | 0.62 | -0.71 | -0.37 | -0.35 | -0.35 | -0.37 | -0.30 | -0.28 | 0.63 | 0.66 | 0.50 | 0.53 | 0.67 | 0.57 |
| ITC1 | 0.52 | 0.83 | 0.19 | -0.88 | 0.41 | -1.03 | -0.16 | -0.91 | -0.92 | 2.93 | -0.69 | -1.27 | -0.12 | -0.21 | -0.28 | -0.60 | -0.23 | -0.20 | 0.57 | 0.71 | 0.46 | 0.53 | 0.64 | 0.53 |
| ITC2 | -0.68 | 0.82 | -0.86 | -0.84 | 0.68 | -0.34 | -0.71 | -1.40 | -0.72 | 0.43 | -0.89 | -0.35 | -0.12 | -0.21 | -0.28 | -0.64 | -0.23 | -0.20 | 0.57 | 0.71 | 0.46 | 0.53 | 0.64 | 0.53 |
| ITC3 | -0.03 | 0.86 | -0.71 | 1.35 | -0.18 | 0.66 | -0.52 | -1.13 | -1.29 | 0.95 | 0.35 | -0.25 | -0.12 | -0.21 | -0.28 | -0.64 | -0.23 | -0.20 | 0.71 | 0.95 | 0.47 | 0.55 | 0.74 | 0.55 |
| ITC4 | 0.71 | 0.99 | -0.25 | 1.35 | 1.06 | -0.76 | 0.50 | -0.13 | -1.60 | 5.28 | -0.84 | -1.64 | -0.07 | -0.13 | -0.28 | -0.34 | -0.23 | -0.20 | 0.78 | 0.97 | 0.47 | 0.59 | 0.82 | 0.67 |
| ITD1 | -0.44 | 0.64 | -0.88 | 0.72 | 0.70 | -1.32 | 0.50 | -0.38 | -0.05 | 0.14 | 0.28 | 0.19 | -0.07 | -0.13 | -0.31 | -0.34 | -0.23 | -0.26 | 0.68 | 0.85 | 0.53 | 0.59 | 0.70 | 0.67 |
| ITD2 | -0.93 | 0.86 | 0.20 | -0.75 | 0.83 | -0.64 | 0.01 | -1.13 | 0.03 | 0.73 | 1.16 | -0.17 | -0.07 | -0.13 | -0.31 | -0.34 | -0.23 | -0.26 | 0.51 | 0.72 | 0.39 | 0.50 | 0.61 | 0.48 |
| ITD3 | -0.06 | 0.89 | -0.14 | 0.49 | 1.04 | -0.80 | 1.52 | -0.76 | -1.20 | 2.88 | -0.69 | -1.26 | -0.07 | -0.13 | -0.31 | -0.34 | -0.23 | -0.26 | 0.62 | 0.77 | 0.45 | 0.58 | 0.72 | 0.65 |
| ITD4 | 0.18 | 0.82 | -0.18 | -0.49 | 0.09 | -0.33 | -0.25 | -0.97 | -0.70 | 0.97 | -0.28 | -0.48 | -0.19 | -0.28 | -0.39 | -0.37 | -0.24 | -0.30 | 0.64 | 0.82 | 0.42 | 0.56 | 0.74 | 0.57 |
| ITD5 | 0.26 | 0.77 | 0.10 | 0.61 | 1.03 | -1.02 | -0.02 | -0.68 | -1.00 | 2.91 | -0.59 | -1.25 | -0.12 | -0.21 | -0.28 | -0.51 | -0.23 | -0.20 | 0.70 | 0.85 | 0.46 | 0.59 | 0.79 | 0.63 |
| ITE1 | 0.27 | 1.00 | -0.96 | 0.37 | 0.07 | -0.41 | 1.72 | -0.60 | -0.60 | 2.17 | 0.49 | -0.40 | -0.15 | -0.21 | -0.38 | -0.51 | -0.26 | -0.28 | 0.69 | 0.91 | 0.49 | 0.58 | 0.73 | 0.58 |
| ITE2 | -0.63 | 0.66 | 0.20 | -0.09 | 0.23 | -0.09 | -0.60 | -1.62 | -0.11 | 1.16 | 0.58 | -0.80 | -0.26 | -0.21 | -0.33 | -0.51 | -0.29 | -0.32 | 0.51 | 0.68 | 0.41 | 0.52 | 0.63 | 0.52 |
| ITE3 | -0.23 | 0.87 | 0.19 | -0.04 | 0.47 | -0.13 | 2.42 | -0.56 | -0.77 | 0.80 | -0.55 | -0.84 | -0.15 | -0.21 | -0.33 | -0.51 | -0.26 | -0.28 | 0.51 | 0.64 | 0.43 | 0.53 | 0.64 | 0.53 |
| ITE4 | 0.18 | 1.03 | -0.81 | -0.23 | -0.72 | 0.60 | -0.13 | 0.08 | -0.38 | 2.40 | 2.47 | -0.36 | -0.26 | -0.21 | -0.28 | -0.51 | -0.28 | -0.32 | 0.64 | 0.91 | 0.41 | 0.51 | 0.68 | 0.49 |
| ITF1 | -0.15 | 0.82 | -0.52 | -0.44 | -0.28 | -0.05 | 0.98 | -1.30 | -0.45 | 0.71 | -0.03 | -0.63 | -0.30 | -0.22 | -0.28 | -0.51 | -0.28 | -0.32 | 0.78 | 0.99 | 0.50 | 0.62 | 0.84 | 0.61 |
| ITF2 | -0.66 | 0.71 | -0.53 | -0.61 | -0.50 | -0.02 | 0.74 | -1.63 | 0.10 | 0.41 | 0.09 | -0.62 | -0.38 | -0.22 | -0.28 | -0.47 | -0.28 | -0.35 | 0.78 | 0.99 | 0.50 | 0.62 | 0.84 | 0.61 |
| ITF3 | -0.94 | 0.82 | -0.92 | 1.12 | -0.43 | 0.47 | 0.86 | -1.46 | -0.02 | 2.65 | 1.99 | -0.99 | -0.38 | -0.22 | -0.14 | -0.25 | -0.28 | -0.35 | 0.54 | 0.74 | 0.45 | 0.51 | 0.60 | 0.43 |
| ITF4 | -0.95 | 0.72 | -0.70 | 0.88 | -0.55 | -0.10 | 0.37 | -1.44 | 0.17 | 2.26 | 1.62 | -0.66 | -0.42 | -0.27 | -0.14 | -0.25 | -0.28 | -0.35 | 0.52 | 0.73 | 0.46 | 0.52 | 0.59 | 0.47 |
| ITF5 | -0.36 | 0.73 | -0.76 | -1.01 | -1.09 | -0.13 | -0.08 | -1.55 | 0.36 | 0.57 | -0.29 | -0.20 | -0.51 | -0.27 | -0.14 | -0.25 | -0.29 | -0.35 | 0.41 | 0.56 | 0.39 | 0.42 | 0.49 | 0.45 |
| ITF6 | -1.12 | 0.79 | -1.12 | 1.25 | -0.93 | 0.71 | -0.16 | -1.75 | 0.64 | 1.39 | 2.04 | -0.51 | -0.54 | -0.27 | -0.14 | -0.25 | -0.29 | -0.40 | 0.46 | 0.66 | 0.50 | 0.53 | 0.54 | 0.49 |
| ITG1 | -0.46 | 0.76 | -1.04 | -0.56 | -1.38 | 1.33 | -0.82 | -1.57 | 0.14 | 2.11 | 0.13 | -0.76 | -0.54 | -0.32 | -0.15 | -0.47 | -0.32 | -0.40 | 0.58 | 0.80 | 0.50 | 0.54 | 0.61 | 0.49 |
| ITG2 | -0.49 | 0.64 | -1.25 | -1.13 | -0.82 | -0.28 | -0.95 | -1.46 | 0.29 | 0.88 | 0.23 | -0.38 | -0.59 | -0.39 | -0.27 | -0.39 | -0.28 | -0.52 | 0.47 | 0.71 | 0.46 | 0.51 | 0.56 | 0.49 |
| LT00 | -0.43 | 0.92 | 0.43 | -1.03 | -0.78 | -1.04 | -1.12 | -0.25 | 0.62 | 0.38 | 0.57 | 0.26 | -0.46 | -0.47 | -0.57 | -0.54 | -0.28 | -0.41 | 0.22 | 0.34 | 0.32 | 0.37 | 0.54 | 0.41 |
| LU00 | 0.39 | -0.49 | -0.57 | -0.53 | 0.55 | 0.86 | -0.71 | 0.66 | -0.70 | 0.47 | -0.72 | -0.47 | -0.04 | 0.00 | -0.42 | -0.49 | -0.19 | -0.13 | 0.80 | 0.95 | 0.32 | 0.32 | 0.58 | 0.68 |
| LV00 | -0.13 | 2.42 | -0.21 | -0.14 | -0.16 | -0.93 | -1.11 | 0.81 | 0.48 | -1.78 | -0.58 | 0.38 | -0.34 | -0.35 | -0.36 | -0.26 | -0.36 | -0.64 | 0.10 | 0.52 | 0.20 | 0.28 | 0.40 | 0.08 |
| MT00 | -0.14 | 0.42 | -0.63 | -0.18 | -0.85 | 0.34 | -1.16 | 0.37 | -0.35 | -1.36 | -1.36 | -0.45 | -0.27 | -0.33 | -0.35 | -0.33 | -0.51 | -0.03 | 0.46 | 0.41 | 0.30 | 0.32 | 0.55 | 0.39 |
| NL11 | -0.05 | -0.44 | 0.19 | -0.78 | 0.43 | 1.48 | -0.02 | 0.01 | -0.33 | 0.08 | 0.41 | 1.36 | -0.17 | -0.24 | -0.31 | -0.26 | -0.28 | -0.48 | 0.49 | 0.43 | 0.24 | 0.31 | 0.50 | 0.79 |
| NL12 | -0.43 | -0.24 | 0.37 | 0.31 | 0.83 | 0.60 | 0.47 | -0.43 | -0.05 | -0.02 | 0.72 | 0.37 | -0.15 | -0.18 | -0.28 | -0.26 | -0.28 | -0.48 | 0.71 | 0.61 | 0.37 | 0.36 | 0.60 | 0.98 |
| NL13 | -0.14 | -0.55 | 0.52 | -0.22 | 0.64 | 0.47 | -0.29 | -0.38 | -0.14 | -0.18 | 0.03 | 0.31 | -0.17 | -0.22 | -0.28 | -0.26 | -0.28 | -0.48 | 0.68 | 0.59 | 0.38 | 0.39 | 0.60 | 0.95 |
| NL21 | -0.35 | -0.55 | 0.44 | -0.76 | 0.94 | 0.10 | 0.61 | -0.12 | -0.20 | -0.21 | 0.50 | 0.21 | -0.15 | -0.18 | -0.26 | -0.27 | -0.29 | -0.51 | 0.67 | 0.65 | 0.34 | 0.40 | 0.62 | 0.87 |
| NL22 | 0.10 | -0.33 | 0.11 | 0.00 | 0.97 | 0.55 | 1.11 | 0.14 | -0.66 | 0.18 | 0.86 | 0.90 | -0.11 | -0.10 | -0.18 | -0.24 | -0.29 | -0.51 | 0.76 | 0.73 | 0.36 | 0.42 | 0.67 | 0.87 |
| NL23 | -0.49 | -0.03 | 0.44 | -0.06 | 1.34 | 0.67 | -0.91 | 0.20 | -0.74 | 0.32 | 0.73 | 0.58 | -0.15 | -0.18 | -0.26 | -0.27 | -0.29 | -0.51 | 0.72 | 0.76 | 0.30 | 0.34 | 0.61 | 0.89 |
| NL31 | 0.86 | -0.17 | -0.41 | 0.59 | 0.71 | 1.52 | 0.01 | 1.09 | -1.34 | 0.04 | 1.12 | 1.72 | -0.15 | -0.17 | -0.26 | -0.27 | -0.29 | -0.51 | 0.75 | 0.73 | 0.33 | 0.37 | 0.65 | 0.94 |
| NL32 | 0.52 | -0.28 | -0.40 | 0.28 | 0.72 | 1.01 | -0.23 | 1.24 | -1.01 | 0.27 | 1.19 | 1.05 | -0.15 | -0.17 | -0.30 | -0.32 | -0.29 | -0.51 | 0.80 | 0.79 | 0.38 | 0.39 | 0.67 | 0.98 |
| NL33 | 0.00 | -0.14 | -0.10 | 0.64 | 0.99 | 1.21 | 0.46 | 0.48 | -1.31 | 0.90 | 1.46 | 0.95 | -0.11 | -0.10 | -0.18 | -0.24 | -0.33 | -0.51 | 0.79 | 0.78 | 0.36 | 0.39 | 0.67 | 0.95 |
| NL34 | -0.90 | -0.22 | 0.43 | 0.75 | 1.26 | 0.44 | 0.08 | -0.61 | -0.45 | 0.18 | 1.22 | 0.17 | -0.11 | -0.10 | -0.18 | -0.24 | -0.34 | -0.61 | 0.73 | 0.69 | 0.37 | 0.37 | 0.62 | 1.00 |
| NL41 | 5.19 | -0.37 | -0.61 | 0.48 | -0.23 | -1.52 | -0.82 | 0.24 | 0.15 | -0.71 | -0.90 | -0.19 | -0.11 | -0.10 | -0.18 | -0.24 | -0.33 | -0.51 | 1.00 | 0.99 | 0.48 | 0.62 | 1.00 | 1.00 |
| NL42 | 0.65 | -0.44 | 0.39 | 0.17 | 0.77 | 0.43 | -0.08 | -0.16 | -0.86 | 0.12 | -0.09 | 0.45 | -0.11 | -0.10 | -0.18 | -0.24 | -0.33 | -0.51 | 0.86 | 0.77 | 0.39 | 0.44 | 0.73 | 0.99 |
| PL11 | -1.08 | -1.01 | 2.28 | 0.76 | -0.89 | -0.73 | 0.43 | 0.14 | 0.63 | 1.16 | 0.08 | 1.35 | -0.42 | -0.47 | -0.29 | -0.28 | -0.11 | -0.37 | 0.13 | 0.20 | 0.30 | 0.39 | 0.41 | 0.31 |
| PL12 | 0.07 | -0.71 | 0.65 | 4.40 | -1.03 | -0.86 | -1.56 | 1.04 | 0.51 | 0.85 | 1.06 | 0.83 | -0.47 | -0.47 | -0.24 | -0.16 | -0.11 | -0.11 | 0.28 | 0.44 | 0.41 | 0.43 | 0.49 | 0.23 |
| PL21 | 0.12 | -1.20 | 0.45 | 1.28 | -1.61 | -1.51 | -0.83 | 0.80 | 1.03 | -0.05 | 0.03 | 0.93 | -0.42 | -0.47 | -0.40 | -0.36 | -0.21 | -0.46 | 0.20 | 0.38 | 0.30 | 0.43 | 0.53 | 0.21 |
| PL22 | -0.98 | -0.49 | 2.11 | 1.01 | -0.72 | 0.21 | -1.15 | -0.57 | -0.48 | 0.17 | -0.29 | -0.48 | -0.42 | -0.47 | -0.40 | -0.28 | -0.18 | -0.37 | 0.29 | 0.43 | 0.33 | 0.43 | 0.49 | 0.47 |
| PL31 | -1.26 | -1.77 | 1.57 | -1.16 | -1.20 | -0.86 | 0.18 | 0.87 | 1.57 | 2.08 | -0.36 | 1.34 | -0.47 | -0.47 | -0.35 | -0.33 | -0.23 | -0.39 | 0.32 | 0.32 | 0.35 | 0.35 | 0.41 | 0.29 |
| PL32 | -0.84 | -1.53 | 1.48 | 0.87 | -1.29 | -0.82 | -0.89 | 0.50 | 1.00 | 0.78 | -1.00 | 0.72 | -0.47 | -0.47 | -0.35 | -0.33 | -0.23 | -0.39 | 0.32 | 0.43 | 0.36 | 0.36 | 0.42 | 0.37 |
| PL33 | -1.34 | -1.11 | 1.63 | -0.44 | -1.30 | -0.51 | 0.04 | 0.17 | 1.18 | 1.15 | 0.10 | 0.91 | -0.42 | -0.47 | -0.35 | -0.28 | -0.21 | -0.37 | 0.25 | 0.22 | 0.36 | 0.49 | 0.60 | 0.64 |
| PL34 | -1.70 | -1.72 | 1.90 | 0.78 | -1.02 | -0.15 | -0.91 | 0.17 | 1.35 | 2.34 | 0.02 | 0.72 | -0.49 | -0.54 | -0.24 | -0.16 | -0.21 | -0.11 | 0.29 | 0.37 | 0.55 | 0.48 | 0.36 | 0.14 |
| PL41 | -0.64 | -1.23 | 1.70 | 3.05 | -0.57 | -1.15 | -0.25 | 0.60 | 0.55 | 0.08 | -0.25 | 0.35 | -0.54 | -0.42 | -0.29 | -0.28 | -0.11 | -0.35 | 0.15 | 0.26 | 0.33 | 0.39 | 0.40 | 0.35 |
| PL42 | -0.92 | -0.84 | 0.82 | 1.12 | -1.12 | -0.32 | -0.88 | 0.28 | 0.06 | -0.64 | 0.43 | 0.05 | -0.54 | -0.59 | -0.40 | -0.31 | -0.27 | -0.35 | 0.15 | 0.31 | 0.45 | 0.35 | 0.31 | 0.13 |
| PL43 | -0.82 | -0.76 | 1.52 | 0.13 | -1.01 | -0.19 | 0.22 | 0.09 | 0.31 | -0.84 | -0.61 | 0.40 | -0.62 | -0.42 | -0.40 | -0.30 | -0.24 | -0.35 | 0.09 | 0.17 | 0.35 | 0.53 | 0.30 | 0.35 |
| PL51 | -0.81 | -0.65 | 1.28 | 0.16 | -0.95 | -0.61 | -1.09 | -0.16 | 0.16 | 0.12 | -0.24 | 0.30 | -0.55 | -0.42 | -0.40 | -0.30 | -0.18 | -0.35 | 0.31 | 0.37 | 0.34 | 0.42 | 0.50 | 0.43 |
| PL52 | -0.98 | -0.82 | 1.23 | -0.28 | -1.00 | -0.44 | -0.33 | 0.05 | 0.30 | 0.13 | -0.90 | 0.30 | -0.42 | -0.42 | -0.42 | -0.28 | -0.18 | -0.37 | 0.29 | 0.40 | 0.38 | 0.38 | 0.46 | 0.36 |
| PL61 | -1.38 | -1.03 | 2.21 | 0.33 | -0.44 | -0.79 | -0.76 | -0.55 | 0.40 | 1.07 | -0.14 | 0.11 | -0.49 | -0.48 | -0.24 | -0.16 | -0.11 | -0.34 | 0.11 | 0.33 | 0.33 | 0.44 | 0.39 | 0.29 |
| PL62 | -0.31 | -1.27 | 0.90 | 1.53 | -1.31 | -0.91 | 0.80 | 0.27 | 0.54 | -1.30 | -0.76 | 0.75 | -0.49 | -0.48 | -0.24 | -0.16 | -0.11 | -0.11 | 0.23 | 0.26 | 0.42 | 0.43 | 0.48 | 0.46 |
| PL63 | -1.38 | -0.87 | 1.73 | 3.54 | -0.23 | -0.12 | -1.48 | -0.68 | 0.19 | 0.09 | 0.06 | 0.03 | -0.49 | -0.48 | -0.24 | -0.16 | -0.11 | -0.34 | 0.34 | 0.43 | 0.39 | 0.46 | 0.50 | 0.32 |
| PT11 | 0.42 | 1.72 | -1.18 | 0.45 | -0.15 | -0.94 | 0.60 | 0.20 | 0.15 | 2.08 | -2.46 | 0.62 | -0.13 | -0.19 | -0.35 | -0.37 | -0.23 | -0.28 | 0.56 | 0.68 | 0.49 | 0.49 | 0.82 | 0.55 |
| PT15 | -0.65 | 1.99 | -2.62 | 0.32 | 0.16 | 0.09 | 1.39 | -0.22 | -0.53 | -0.70 | -1.23 | 0.79 | -0.19 | -0.08 | -0.34 | -0.41 | -0.27 | -0.42 | 0.64 | 0.73 | 0.58 | 0.42 | 0.49 | 0.74 |
| PT16 | -0.20 | 1.09 | -1.36 | -0.90 | 0.58 | -1.99 | 2.10 | 0.32 | 0.61 | 2.47 | -1.71 | 0.60 | -0.11 | -0.07 | -0.34 | -0.37 | -0.23 | -0.15 | 0.81 | 0.97 | 0.56 | 0.54 | 0.39 | 0.36 |
| PT17 | 0.10 | 2.36 | -1.19 | 0.72 | 0.30 | 1.39 | -0.89 | -0.25 | -2.75 | 1.69 | -0.76 | 0.34 | -0.11 | -0.07 | -0.34 | -0.37 | -0.23 | -0.15 | 0.76 | 1.00 | 0.50 | 0.47 | 0.61 | 0.57 |
| PT18 | 0.02 | 1.59 | -1.58 | 0.53 | -0.31 | -0.09 | 1.04 | -0.46 | 0.40 | 0.53 | -2.16 | 1.11 | -0.11 | -0.07 | -0.34 | -0.37 | -0.23 | -0.15 | 0.60 | 0.82 | 0.43 | 0.45 | 0.41 | 0.78 |
| PT20 | -0.45 | 1.51 | -2.46 | -1.01 | -0.36 | -0.50 | -0.34 | -0.47 | 0.89 | -0.50 | -0.93 | -0.13 | -0.20 | -0.20 | -0.39 | -0.44 | -0.40 | -0.09 | 0.56 | 0.79 | 0.46 | 0.45 | 0.28 | 0.31 |
| PT30 | -0.46 | 1.72 | -2.75 | -0.87 | -0.40 | -0.51 | -0.47 | -0.44 | 0.33 | -1.08 | -0.67 | -0.20 | -0.20 | -0.20 | -0.39 | -0.44 | -0.40 | -0.09 | 0.56 | 0.79 | 0.46 | 0.45 | 0.28 | 0.31 |
| RO11 | -0.39 | 0.60 | 0.87 | 0.44 | -1.20 | -1.07 | 0.98 | 2.94 | 1.34 | 0.44 | -0.26 | 0.61 | -0.62 | -0.41 | -0.32 | -0.30 | -0.20 | -0.24 | 0.22 | 0.37 | 0.40 | 0.42 | 0.43 | 0.72 |
| RO12 | 0.11 | 1.16 | 0.98 | 0.52 | -1.15 | -0.43 | 2.01 | 2.63 | 0.35 | -0.32 | -1.56 | 0.21 | -0.30 | -0.31 | -0.32 | -0.30 | -0.20 | -0.19 | 0.19 | 0.34 | 0.42 | 0.56 | 0.46 | 0.48 |
| RO21 | -0.88 | -0.01 | 1.01 | 0.25 | -1.53 | 0.71 | 1.25 | 2.63 | 1.74 | 3.59 | -1.40 | 1.36 | -0.30 | -0.31 | -0.32 | -0.32 | -0.33 | -0.19 | 0.34 | 0.55 | 0.47 | 0.45 | 0.47 | 0.75 |
| RO22 | -0.91 | 0.21 | 1.09 | 0.89 | -1.04 | -1.14 | -0.81 | 1.76 | 1.40 | 1.62 | -0.41 | 0.93 | -0.30 | -0.31 | -0.32 | -0.33 | -0.33 | -0.19 | 0.74 | 0.60 | 0.56 | 0.53 | 0.46 | 0.99 |
| RO31 | -0.37 | 0.33 | 1.02 | 0.33 | -1.17 | -0.51 | 3.02 | 1.85 | 1.22 | 2.40 | -2.34 | 1.61 | -0.30 | -0.31 | -0.32 | -0.31 | -0.24 | -0.19 | 0.18 | 0.32 | 0.40 | 0.43 | 0.56 | 0.43 |
| RO32 | -1.04 | 2.08 | 0.81 | -0.48 | 0.20 | -0.36 | -0.72 | 7.38 | -1.00 | -0.88 | 2.28 | -1.27 | -0.82 | -0.37 | -0.37 | -0.36 | -0.34 | -0.21 | 0.07 | 0.80 | 0.35 | 0.42 | 0.40 | 0.55 |
| RO41 | -0.47 | -0.19 | 0.97 | -0.08 | -2.03 | 0.51 | 0.57 | 2.75 | 1.88 | 1.99 | -1.46 | 1.11 | -0.74 | -0.44 | -0.32 | -0.30 | -0.20 | -0.26 | 0.03 | 0.33 | 0.56 | 0.52 | 0.34 | 0.36 |
| RO42 | -0.11 | 0.79 | 1.08 | 0.59 | -1.15 | -0.59 | -0.22 | 2.93 | 0.53 | 0.14 | -1.33 | -0.31 | -0.74 | -0.44 | -0.32 | -0.30 | -0.20 | -0.24 | 0.00 | 0.16 | 0.30 | 0.43 | 0.41 | 0.53 |
| SE11 | 2.68 | 1.32 | -0.64 | 1.38 | 1.91 | 1.11 | -0.67 | 0.53 | 3.19 | 0.34 | 1.67 | -0.16 | -0.08 | -0.07 | -0.25 | -0.18 | -0.21 | -0.31 | 1.00 | 1.00 | 0.44 | 0.52 | 0.92 | 0.94 |
| SE12 | 0.97 | 0.51 | 0.43 | -1.05 | 1.97 | 0.83 | 1.16 | -1.30 | 3.53 | 0.70 | 0.52 | -0.27 | -0.07 | -0.07 | -0.24 | -0.18 | -0.21 | -0.31 | 0.82 | 0.76 | 0.36 | 0.46 | 0.74 | 0.81 |
| SE21 | -0.36 | 0.29 | 0.85 | 0.47 | 2.52 | 1.24 | 1.52 | -0.30 | 2.68 | -0.18 | -1.06 | -0.59 | -0.07 | -0.08 | -0.23 | -0.18 | -0.21 | -0.31 | 0.65 | 0.52 | 0.34 | 0.38 | 0.60 | 0.91 |
| SE22 | 2.38 | 0.53 | -0.12 | 0.11 | 1.46 | 0.64 | -0.63 | -0.73 | 3.68 | 0.05 | -0.10 | -0.18 | -0.07 | -0.08 | -0.23 | -0.20 | -0.23 | -0.35 | 1.00 | 0.89 | 0.44 | 0.50 | 0.83 | 0.97 |
| SE23 | 1.65 | 0.83 | 0.39 | 0.39 | 2.19 | 0.65 | -0.61 | -0.68 | 3.18 | 0.15 | -0.59 | -0.75 | -0.07 | -0.08 | -0.23 | -0.18 | -0.21 | -0.31 | 1.00 | 0.90 | 0.41 | 0.48 | 0.83 | 0.96 |
| SE31 | -0.15 | 0.31 | 0.54 | 0.60 | 1.86 | 1.57 | 1.19 | -0.39 | 2.87 | -0.53 | -0.61 | -0.73 | -0.07 | -0.09 | -0.24 | -0.18 | -0.21 | -0.31 | 0.78 | 0.68 | 0.42 | 0.45 | 0.68 | 0.94 |
| SE32 | -0.74 | 0.16 | 0.50 | 1.24 | 1.89 | 2.01 | -0.41 | -0.66 | 2.99 | -0.20 | -0.20 | -0.52 | -0.13 | -0.19 | -0.25 | -0.16 | -0.21 | -0.30 | 0.62 | 0.55 | 0.35 | 0.37 | 0.56 | 0.85 |
| SE33 | -0.05 | -0.07 | 0.44 | -0.99 | 1.37 | 2.21 | -0.42 | -1.66 | 4.05 | 0.67 | 0.65 | 0.05 | -0.15 | -0.20 | -0.25 | -0.16 | -0.21 | -0.30 | 0.62 | 0.59 | 0.35 | 0.44 | 0.61 | 0.71 |
| SI01 | 0.23 | 2.62 | 1.59 | -0.40 | -0.06 | -0.28 | -0.03 | 0.44 | 0.40 | -0.66 | -0.85 | 0.04 | -0.48 | -0.25 | -0.21 | -0.24 | -0.23 | -0.28 | 0.45 | 0.52 | 0.37 | 0.47 | 0.50 | 0.57 |
| SI02 | 0.38 | 2.91 | 1.26 | 0.47 | 0.15 | 0.10 | -0.86 | 0.29 | -0.06 | -0.65 | 1.18 | 0.16 | -0.48 | -0.25 | -0.21 | -0.24 | -0.23 | -0.28 | 0.54 | 0.66 | 0.33 | 0.43 | 0.48 | 0.42 |
| SK01 | -0.10 | 4.11 | 0.71 | -0.87 | -0.14 | 0.65 | -0.21 | 0.92 | -0.18 | -0.53 | 3.27 | -0.03 | -0.57 | -0.60 | -0.72 | -0.46 | -0.26 | -0.27 | 0.37 | 0.48 | 0.15 | 0.30 | 0.53 | 0.47 |
| SK02 | 0.17 | 3.91 | 1.60 | 0.14 | -0.47 | 0.21 | 1.29 | -0.46 | -0.12 | -0.57 | -0.16 | -1.00 | -0.57 | -0.60 | -0.64 | -0.43 | -0.26 | -0.27 | 0.22 | 0.37 | 0.30 | 0.42 | 0.41 | 0.16 |
| SK03 | -0.58 | 4.10 | 1.41 | -1.40 | -0.81 | 0.87 | 0.81 | -0.75 | 0.36 | 0.15 | 0.65 | 0.00 | -0.57 | -0.74 | -0.64 | -0.43 | -0.29 | -0.44 | 0.30 | 0.36 | 0.26 | 0.42 | 0.47 | 0.27 |
| SK04 | -0.21 | 3.90 | 1.07 | -0.88 | -1.24 | 0.84 | -0.25 | -0.80 | 0.14 | -0.33 | 0.28 | -0.61 | -0.80 | -0.74 | -0.64 | -0.43 | -0.29 | -0.44 | 0.20 | 0.37 | 0.20 | 0.43 | 0.38 | 0.45 |
| UKC1 | -0.78 | -0.47 | -0.26 | -0.65 | 0.94 | 0.90 | 0.43 | 0.13 | 0.08 | -0.46 | -0.91 | -0.48 | -0.12 | -0.24 | -0.17 | -0.25 | -0.26 | -0.31 | 0.54 | 0.56 | 0.52 | 0.53 | 0.60 | 0.68 |
| UKC2 | -0.96 | -0.55 | -0.28 | -0.68 | 0.90 | 1.24 | -0.04 | 0.06 | 0.34 | -0.30 | -0.65 | -0.42 | -0.19 | -0.24 | -0.17 | -0.25 | -0.27 | -0.28 | 0.54 | 0.56 | 0.52 | 0.53 | 0.60 | 0.68 |
| UKD1 | -0.42 | -0.37 | -0.26 | 0.40 | 1.35 | -0.21 | -0.25 | 0.49 | -0.09 | -1.03 | -1.18 | -0.31 | -0.12 | -0.24 | -0.17 | -0.25 | -0.26 | -0.28 | 0.57 | 0.47 | 0.52 | 0.52 | 0.66 | 0.69 |
| UKD2 | 0.56 | -0.22 | -0.12 | 0.40 | 1.03 | 0.25 | -0.51 | 1.28 | -0.30 | -0.43 | -0.59 | -0.30 | -0.15 | -0.24 | -0.17 | -0.25 | -0.27 | -0.28 | 0.57 | 0.47 | 0.52 | 0.52 | 0.66 | 0.69 |
| UKD3 | -0.29 | -0.14 | -0.62 | -0.22 | 0.94 | 1.32 | -0.11 | 0.53 | -1.27 | 0.56 | -1.01 | 0.22 | -0.12 | -0.24 | -0.23 | -0.25 | -0.26 | -0.31 | 0.57 | 0.47 | 0.52 | 0.52 | 0.66 | 0.69 |
| UKD4 | 0.09 | -0.26 | -0.31 | -0.07 | 0.97 | 0.94 | -0.30 | 0.42 | -0.27 | -0.36 | -1.39 | -0.51 | -0.12 | -0.24 | -0.23 | -0.25 | -0.26 | -0.31 | 0.57 | 0.47 | 0.52 | 0.52 | 0.66 | 0.69 |
| UKD5 | -0.67 | -0.38 | -0.37 | -0.87 | 0.53 | 1.51 | -0.78 | 0.06 | 0.26 | 0.11 | -0.48 | -0.43 | -0.19 | -0.24 | -0.23 | -0.25 | -0.29 | -0.37 | 0.57 | 0.47 | 0.52 | 0.52 | 0.66 | 0.69 |
| UKE1 | -0.94 | -0.29 | -0.22 | -0.71 | 1.11 | 0.44 | -0.35 | 0.25 | 0.00 | -0.51 | -0.81 | -0.53 | -0.12 | -0.28 | -0.23 | -0.32 | -0.26 | -0.31 | 0.48 | 0.48 | 0.54 | 0.42 | 0.54 | 0.61 |
| UKE2 | -0.71 | -0.40 | -0.49 | -0.12 | 1.32 | 0.44 | -0.42 | 0.55 | 0.45 | 0.01 | 0.61 | 0.31 | -0.12 | -0.24 | -0.17 | -0.25 | -0.26 | -0.31 | 0.48 | 0.48 | 0.54 | 0.42 | 0.54 | 0.61 |
| UKE3 | -0.95 | -0.33 | -0.35 | -0.62 | 1.17 | 0.73 | -0.20 | -0.25 | -0.12 | 0.17 | 0.05 | -0.13 | -0.12 | -0.24 | -0.23 | -0.25 | -0.26 | -0.31 | 0.48 | 0.48 | 0.54 | 0.42 | 0.54 | 0.61 |
| UKE4 | -0.83 | -0.34 | -0.39 | -0.40 | 1.53 | 0.19 | -0.28 | 0.49 | -0.52 | 0.20 | -0.29 | -0.25 | -0.12 | -0.24 | -0.23 | -0.25 | -0.26 | -0.31 | 0.48 | 0.48 | 0.54 | 0.42 | 0.54 | 0.61 |
| UKF1 | -0.53 | -0.37 | -0.02 | 0.23 | 1.36 | 0.35 | -0.15 | 0.19 | 0.13 | 0.08 | -0.10 | -0.55 | -0.12 | -0.24 | -0.17 | -0.25 | -0.26 | -0.31 | 0.64 | 0.55 | 0.47 | 0.38 | 0.60 | 0.75 |
| UKF2 | -0.82 | -0.58 | 0.04 | -0.03 | 2.02 | -0.32 | 0.33 | 0.48 | -0.17 | -0.03 | -0.22 | -0.62 | -0.15 | -0.21 | -0.17 | -0.27 | -0.26 | -0.31 | 0.64 | 0.55 | 0.47 | 0.38 | 0.60 | 0.75 |
| UKF3 | -1.01 | -0.61 | -0.18 | -0.25 | 1.44 | -0.05 | -0.14 | 0.51 | 0.12 | -0.69 | -1.00 | -0.34 | -0.12 | -0.28 | -0.23 | -0.28 | -0.26 | -0.31 | 0.64 | 0.55 | 0.47 | 0.38 | 0.60 | 0.75 |
| UKG1 | 0.01 | -0.53 | -0.22 | -0.56 | 1.37 | -0.08 | 0.97 | 0.90 | -0.31 | -0.68 | -1.32 | -0.62 | -0.15 | -0.21 | -0.17 | -0.27 | -0.26 | -0.31 | 0.59 | 0.48 | 0.58 | 0.48 | 0.59 | 0.61 |
| UKG2 | -0.66 | -0.43 | -0.30 | -0.31 | 1.32 | 0.00 | 0.43 | 0.64 | -0.14 | -0.69 | -1.09 | -0.47 | -0.15 | -0.24 | -0.17 | -0.25 | -0.26 | -0.26 | 0.59 | 0.48 | 0.58 | 0.48 | 0.59 | 0.61 |
| UKG3 | -0.46 | -0.50 | -0.28 | -0.82 | 0.96 | 0.56 | -0.67 | 0.65 | -0.68 | 0.50 | -0.64 | -0.49 | -0.20 | -0.35 | -0.17 | -0.27 | -0.28 | -0.31 | 0.59 | 0.48 | 0.58 | 0.48 | 0.59 | 0.61 |
| UKH1 | 1.59 | -0.21 | -0.77 | -0.28 | 0.69 | -0.47 | -0.53 | 0.43 | 1.03 | 0.05 | 0.70 | 0.47 | -0.15 | -0.28 | -0.24 | -0.28 | -0.28 | -0.33 | 0.68 | 0.60 | 0.49 | 0.50 | 0.77 | 0.71 |
| UKH2 | 0.60 | -0.10 | -0.57 | -0.13 | 1.73 | -0.41 | 0.70 | 1.18 | -0.41 | -0.25 | -0.16 | -0.22 | -0.15 | -0.21 | -0.24 | -0.28 | -0.26 | -0.33 | 0.68 | 0.60 | 0.49 | 0.50 | 0.77 | 0.71 |
| UKH3 | 0.19 | -0.27 | -0.49 | -0.47 | 1.75 | -0.78 | 0.58 | 0.71 | -0.12 | -0.19 | -0.79 | -0.83 | -0.15 | -0.21 | -0.24 | -0.28 | -0.26 | -0.40 | 0.68 | 0.60 | 0.49 | 0.50 | 0.77 | 0.71 |
| UKI1 | -0.57 | -0.69 | -0.25 | -1.48 | 1.84 | 1.73 | -0.42 | 6.13 | -0.53 | 2.71 | 1.24 | -0.57 | -0.15 | -0.28 | -0.28 | -0.37 | -0.26 | -0.43 | 0.38 | 0.36 | 0.32 | 0.37 | 0.57 | 0.75 |
| UKI2 | -0.83 | -0.15 | -0.67 | 0.32 | 1.71 | 0.64 | -0.14 | 2.02 | -0.60 | 0.76 | 0.89 | -0.52 | -0.15 | -0.21 | -0.24 | -0.28 | -0.26 | -0.40 | 0.38 | 0.36 | 0.32 | 0.37 | 0.57 | 0.75 |
| UKJ1 | 0.61 | -0.28 | -0.27 | 0.47 | 1.80 | -0.36 | -0.36 | 1.77 | -0.20 | 0.72 | 1.42 | 0.17 | -0.15 | -0.21 | -0.17 | -0.27 | -0.26 | -0.31 | 0.58 | 0.63 | 0.57 | 0.39 | 0.59 | 0.56 |
| UKJ2 | 0.42 | -0.55 | -0.39 | -0.68 | 1.45 | -0.14 | -0.40 | 1.78 | 0.06 | 0.43 | 0.04 | -0.46 | -0.15 | -0.21 | -0.28 | -0.33 | -0.26 | -0.40 | 0.58 | 0.63 | 0.57 | 0.39 | 0.59 | 0.56 |
| UKJ3 | 0.71 | -0.43 | -0.44 | 0.10 | 1.33 | -0.22 | -1.05 | 0.87 | 0.18 | -0.33 | 0.32 | -0.61 | -0.15 | -0.21 | -0.23 | -0.33 | -0.26 | -0.35 | 0.58 | 0.63 | 0.57 | 0.39 | 0.59 | 0.56 |
| UKJ4 | -0.44 | -0.35 | -0.40 | -0.15 | 1.64 | 0.17 | -0.41 | 0.69 | -0.39 | -0.12 | -0.52 | -0.99 | -0.15 | -0.21 | -0.24 | -0.28 | -0.26 | -0.40 | 0.58 | 0.63 | 0.57 | 0.39 | 0.59 | 0.56 |
| UKK1 | 0.62 | -0.32 | -0.43 | 0.37 | 1.44 | -0.47 | 0.01 | 0.93 | 0.16 | 0.12 | 0.59 | -0.27 | -0.15 | -0.21 | -0.17 | -0.27 | -0.26 | -0.26 | 0.62 | 0.53 | 0.48 | 0.43 | 0.63 | 0.73 |
| UKK2 | -0.73 | -0.70 | -0.56 | -0.59 | 1.41 | 0.03 | -0.03 | 0.72 | 0.15 | -0.71 | -0.86 | -0.53 | -0.15 | -0.21 | -0.23 | -0.33 | -0.26 | -0.35 | 0.62 | 0.53 | 0.48 | 0.43 | 0.63 | 0.73 |
| UKK3 | -0.96 | -0.63 | -0.42 | -0.48 | 1.20 | 0.26 | -0.85 | 0.47 | 0.17 | -1.09 | -1.22 | -0.37 | -0.18 | -0.22 | -0.23 | -0.34 | -0.27 | -0.35 | 0.62 | 0.53 | 0.48 | 0.43 | 0.63 | 0.73 |
| UKK4 | -1.04 | -0.68 | -0.57 | -0.61 | 1.02 | 0.63 | -0.94 | 0.53 | 0.19 | -0.67 | -0.70 | -0.32 | -0.18 | -0.22 | -0.23 | -0.34 | -0.27 | -0.35 | 0.62 | 0.53 | 0.48 | 0.43 | 0.63 | 0.73 |
| UKL1 | -0.96 | -0.75 | -0.49 | -0.76 | 0.71 | 0.51 | -0.81 | 0.09 | 0.39 | -0.50 | -0.65 | -0.34 | -0.18 | -0.33 | -0.21 | -0.28 | -0.26 | -0.26 | 0.61 | 0.52 | 0.53 | 0.49 | 0.59 | 0.70 |
| UKL2 | -0.62 | -0.50 | -0.45 | -0.55 | 1.29 | 0.25 | -0.03 | 0.61 | 0.32 | -0.10 | 0.20 | 0.11 | -0.18 | -0.24 | -0.17 | -0.25 | -0.26 | -0.26 | 0.61 | 0.52 | 0.53 | 0.49 | 0.59 | 0.70 |
| UKM2 | -0.42 | -0.54 | -0.39 | -0.07 | 1.19 | 0.21 | -0.74 | 0.51 | 0.91 | 0.26 | 1.75 | 0.02 | -0.19 | -0.24 | -0.17 | -0.25 | -0.27 | -0.28 | 0.49 | 0.57 | 0.49 | 0.46 | 0.55 | 0.64 |
| UKM3 | -1.15 | -0.57 | -0.53 | 0.12 | 1.07 | 0.77 | -0.70 | 0.45 | 0.26 | -0.05 | 0.76 | -0.44 | -0.19 | -0.24 | -0.17 | -0.25 | -0.27 | -0.28 | 0.49 | 0.57 | 0.49 | 0.46 | 0.55 | 0.64 |
| UKM5 | -0.73 | -0.83 | 0.01 | -1.05 | 1.39 | -0.90 | -0.81 | 1.24 | 0.33 | -0.69 | 0.93 | 0.01 | -0.19 | -0.24 | -0.27 | -0.33 | -0.27 | -0.28 | 0.49 | 0.57 | 0.49 | 0.46 | 0.55 | 0.64 |
| UKM6 | -0.92 | -0.74 | -0.43 | -0.62 | 1.20 | 0.22 | -0.70 | 0.44 | 0.54 | -1.40 | -0.60 | 0.21 | -0.19 | -0.24 | -0.27 | -0.33 | -0.27 | -0.28 | 0.49 | 0.57 | 0.49 | 0.46 | 0.55 | 0.64 |
| UKN0 | -0.75 | -0.60 | -0.82 | -1.07 | 0.27 | 0.60 | -0.53 | -0.15 | 0.49 | 0.00 | -0.31 | -0.17 | -0.11 | -0.33 | -0.24 | -0.28 | -0.34 | -0.20 | 0.43 | 0.38 | 0.44 | 0.50 | 0.52 | 0.59 |
