# Supplementary material for: Modelling innovation performance of European regions using multi-output neural networks
Source: PLoS One. 2017 Oct 2;12(10):e0185755. doi: 10.1371/journal.pone.0185755 (PMC5624612; doi:10.1371/journal.pone.0185755)
Supplement: S2 Appendix — (DOCX) [file pone.0185755.s002.docx]

S2 Appendix - Component loadings

|  | *CP*_1_ | *CP*_2_ | *CP*_3_ | *CP* _4_ | *CP*_5_ | *CP*_6_ | *CP*_7_ | *CP*_8_ | *CP*_9_ | *CP*_10_ | *CP*_11_ | *CP*_12_ |
| --- | --- | --- | --- | --- | --- | --- | --- | --- | --- | --- | --- | --- |
| *x*_1_ | 0.446 | -0.276 | -0.222 | -0.161 | 0.547 | 0.256 | -0.027 | 0.045 | -0.216 | 0.144 | 0.173 | 0.011 |
| *x*_2_ | 0.265 | 0.009 | -0.019 | -0.134 | 0.787 | -0.029 | -0.055 | 0.227 | 0.054 | -0.063 | -0.025 | 0.102 |
| *x*_3_ | -0.034 | 0.157 | 0.280 | 0.038 | -0.813 | 0.023 | 0.085 | -0.020 | -0.101 | 0.093 | 0.136 | 0.023 |
| *x*_4_ | -0.284 | -0.040 | 0.099 | 0.061 | -0.514 | -0.336 | 0.045 | 0.190 | 0.415 | 0.299 | -0.231 | 0.214 |
| *x*_5_ | 0.149 | 0.180 | 0.676 | 0.063 | -0.078 | -0.303 | 0.247 | -0.091 | -0.121 | -0.070 | -0.264 | -0.215 |
| *x*_6_ | -0.312 | 0.143 | -0.590 | 0.032 | 0.062 | -0.343 | 0.092 | -0.310 | -0.090 | -0.185 | 0.091 | -0.109 |
| *x*_7_ | -0.189 | 0.104 | -0.535 | -0.013 | 0.198 | -0.117 | -0.351 | -0.057 | -0.352 | -0.265 | 0.116 | -0.092 |
| *x*_8_ | 0.413 | -0.118 | -0.148 | 0.015 | 0.493 | 0.297 | -0.154 | 0.193 | -0.294 | 0.172 | 0.385 | -0.004 |
| *x*_9_ | 0.125 | -0.203 | -0.198 | -0.143 | 0.265 | 0.770 | -0.048 | -0.146 | 0.060 | -0.194 | 0.207 | 0.069 |
| *x*_10_ | 0.044 | -0.058 | 0.003 | -0.040 | 0.134 | 0.816 | -0.091 | 0.105 | -0.026 | -0.070 | -0.086 | 0.011 |
| *x*_11_ | 0.074 | -0.064 | 0.006 | -0.078 | 0.288 | 0.037 | -0.092 | -0.044 | -0.082 | -0.120 | 0.091 | 0.750 |
| *x*_12_ | 0.023 | 0.090 | 0.850 | -0.011 | -0.076 | -0.083 | 0.010 | -0.021 | 0.015 | -0.162 | 0.258 | -0.002 |
| *x*_13_ | 0.273 | -0.054 | 0.034 | -0.088 | 0.771 | 0.262 | -0.087 | -0.008 | 0.299 | -0.041 | 0.151 | 0.236 |
| *x*_14_ | 0.526 | -0.198 | 0.024 | -0.046 | 0.466 | 0.242 | -0.057 | 0.115 | -0.172 | -0.146 | 0.431 | 0.173 |
| *x*_15_ | 0.328 | -0.048 | 0.052 | 0.006 | -0.055 | -0.012 | -0.141 | 0.081 | -0.091 | -0.007 | 0.691 | 0.070 |
| *x*_16_ | 0.375 | -0.156 | -0.077 | -0.223 | 0.259 | 0.209 | -0.100 | -0.244 | 0.178 | 0.185 | 0.405 | 0.195 |
| *x*_17_ | 0.320 | -0.289 | 0.035 | -0.000 | 0.651 | 0.386 | -0.040 | 0.031 | -0.083 | -0.124 | 0.043 | 0.237 |
| *x*_18_ | -0.043 | 0.053 | 0.051 | -0.062 | 0.139 | 0.042 | -0.061 | 0.766 | -0.063 | 0.104 | 0.121 | -0.053 |
| *x*_19_ | 0.041 | -0.280 | -0.242 | -0.044 | 0.658 | 0.308 | -0.023 | 0.243 | 0.282 | -0.044 | -0.094 | -0.028 |
| *x*_20_ | 0.355 | -0.055 | -0.090 | 0.022 | 0.160 | 0.278 | -0.022 | -0.017 | -0.568 | 0.136 | 0.110 | 0.122 |
| *x*_21_ | 0.764 | -0.140 | 0.118 | -0.046 | 0.316 | 0.050 | -0.052 | -0.050 | 0.083 | -0.012 | 0.044 | -0.092 |
| *x*_22_ | -0.322 | 0.302 | 0.407 | 0.077 | -0.073 | -0.109 | 0.049 | -0.228 | 0.087 | 0.328 | -0.327 | 0.321 |
| *x*_23_ | 0.182 | 0.017 | 0.021 | -0.036 | 0.732 | 0.021 | -0.090 | -0.206 | -0.179 | 0.121 | -0.063 | 0.131 |
| *x*_24_ | -0.145 | 0.913 | 0.006 | -0.051 | -0.194 | -0.123 | -0.028 | -0.049 | 0.025 | 0.143 | -0.052 | -0.012 |
| *x*_25_ | -0.081 | 0.828 | 0.164 | -0.118 | -0.113 | 0.021 | 0.012 | -0.025 | 0.015 | 0.061 | 0.113 | -0.026 |
| *x*_26_ | -0.138 | 0.894 | 0.138 | -0.108 | -0.162 | -0.044 | -0.036 | -0.012 | -0.021 | 0.074 | 0.043 | -0.005 |
| *x*_27_ | -0.056 | 0.870 | -0.019 | 0.099 | 0.241 | -0.061 | -0.049 | -0.163 | -0.049 | -0.031 | -0.021 | -0.105 |
| *x*_28_ | -0.078 | 0.696 | -0.192 | 0.025 | 0.016 | -0.055 | -0.163 | 0.270 | 0.024 | -0.283 | -0.157 | 0.010 |
| *x*_29_ | 0.073 | 0.051 | -0.093 | 0.228 | -0.081 | -0.227 | 0.063 | 0.138 | -0.083 | 0.742 | 0.056 | -0.116 |
| *x*_30_ | 0.838 | -0.129 | 0.008 | -0.016 | 0.161 | -0.007 | -0.078 | 0.014 | 0.138 | -0.003 | 0.097 | 0.066 |
| *x*_31_ | 0.584 | -0.010 | -0.043 | -0.069 | 0.122 | 0.177 | 0.010 | -0.014 | -0.107 | 0.077 | 0.137 | 0.437 |
| *x*_32_ | 0.852 | -0.211 | 0.150 | -0.095 | 0.213 | 0.001 | 0.030 | -0.067 | -0.095 | 0.007 | 0.014 | -0.039 |
| *x*_33a_ | -0.252 | -0.053 | -0.023 | -0.075 | -0.330 | -0.195 | -0.025 | 0.124 | -0.737 | 0.019 | -0.031 | 0.082 |
| *x*_33b_ | -0.364 | 0.497 | -0.096 | 0.296 | -0.434 | -0.340 | -0.083 | -0.017 | 0.042 | 0.192 | -0.143 | -0.029 |
| *x*_33c_ | -0.091 | 0.680 | 0.069 | -0.009 | -0.279 | -0.067 | 0.064 | 0.349 | 0.084 | -0.097 | -0.201 | 0.072 |
| *x*_34_ | 0.020 | -0.014 | -0.078 | 0.201 | -0.207 | 0.114 | 0.762 | 0.055 | -0.034 | 0.063 | -0.059 | 0.090 |
| *x*_35_ | -0.107 | -0.036 | 0.028 | 0.218 | 0.001 | -0.091 | 0.806 | -0.072 | -0.015 | -0.004 | 0.031 | -0.076 |
| *x*_36_ | 0.015 | -0.023 | 0.085 | 0.696 | -0.236 | -0.092 | 0.195 | 0.085 | 0.155 | -0.105 | -0.137 | 0.077 |
| *x*_37_ | 0.092 | -0.034 | -0.078 | 0.710 | -0.109 | -0.055 | 0.188 | 0.009 | -0.021 | -0.023 | -0.142 | -0.009 |
| *x*_38_ | -0.252 | -0.049 | 0.126 | 0.695 | 0.072 | 0.010 | 0.072 | -0.047 | -0.047 | 0.198 | 0.214 | -0.099 |
| *x*_39_ | -0.166 | -0.038 | -0.012 | 0.717 | 0.013 | -0.032 | -0.033 | -0.165 | -0.019 | 0.248 | 0.270 | -0.100 |
| *x*_40_ | -0.079 | 0.018 | -0.009 | 0.285 | -0.156 | -0.025 | 0.406 | 0.079 | 0.040 | 0.059 | 0.535 | 0.053 |
| *x*_41_ | -0.035 | -0.102 | 0.186 | -0.062 | 0.002 | -0.176 | 0.690 | -0.075 | 0.083 | -0.005 | -0.039 | -0.117 |
| *x*_42_ | -0.264 | 0.418 | 0.541 | 0.209 | -0.349 | -0.183 | -0.098 | 0.276 | 0.135 | -0.111 | -0.064 | 0.074 |
