# Supplementary figures and images for: Modelling innovation performance of European regions using multi-output neural networks
Source: PLoS One. 2017 Oct 2;12(10):e0185755. doi: 10.1371/journal.pone.0185755 (PMC5624612; doi:10.1371/journal.pone.0185755)

S3 Appendix – Sensitivity analysis of multi-output ANN


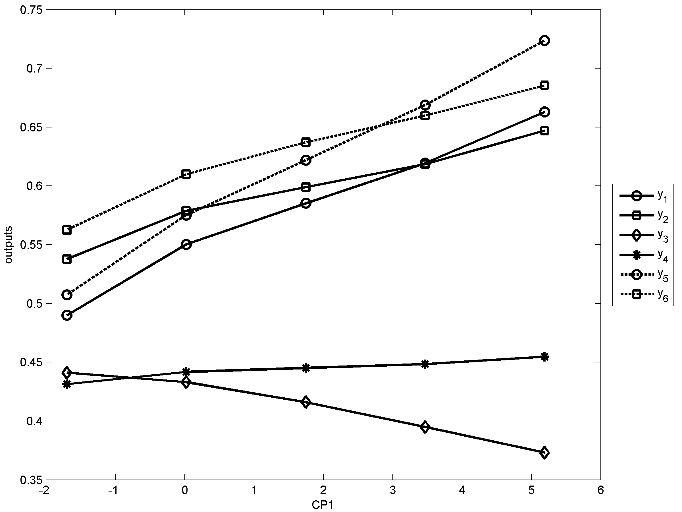

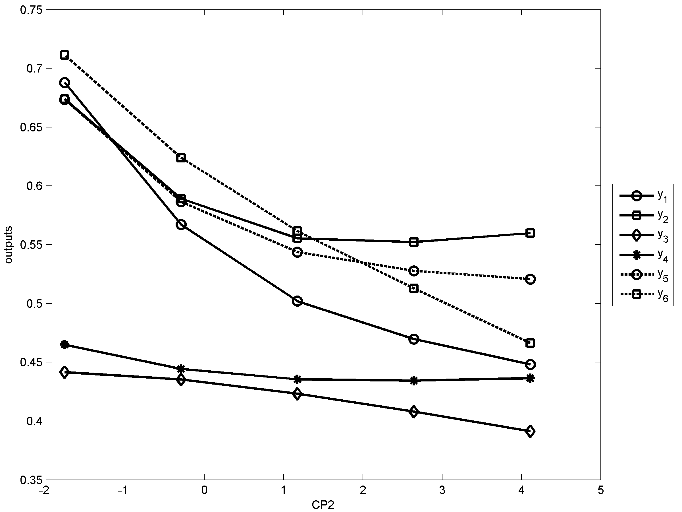

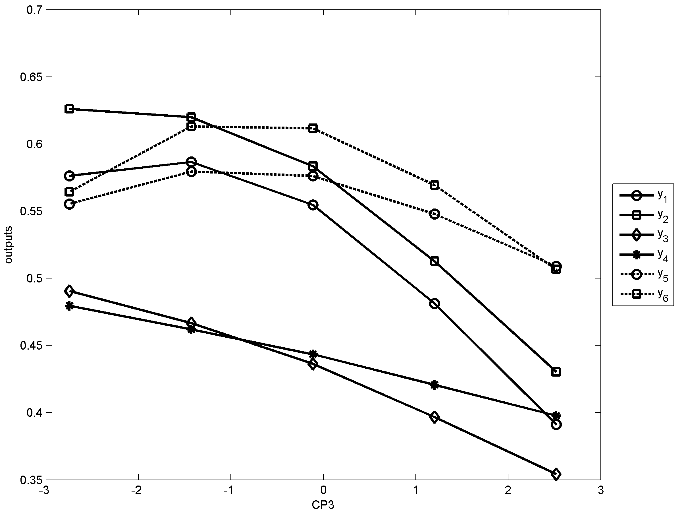

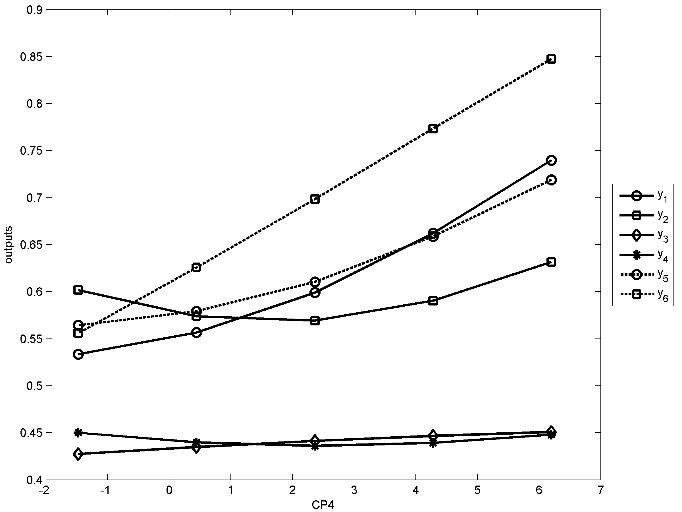

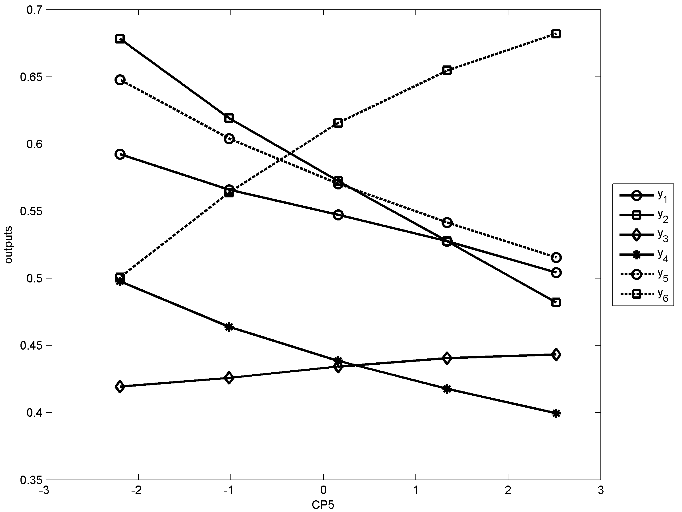

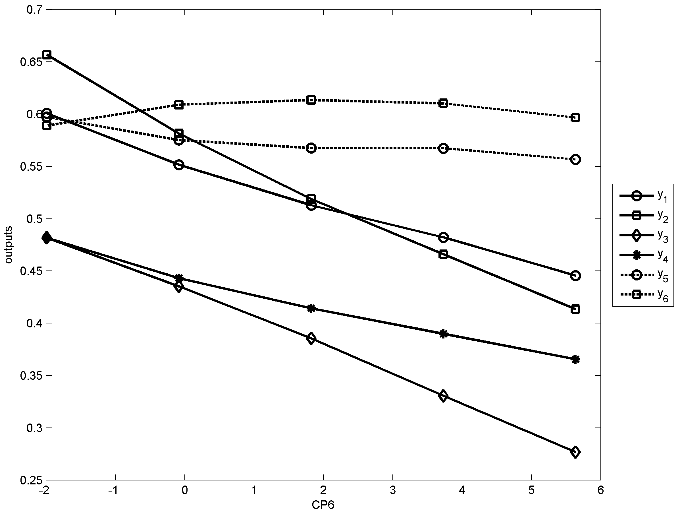

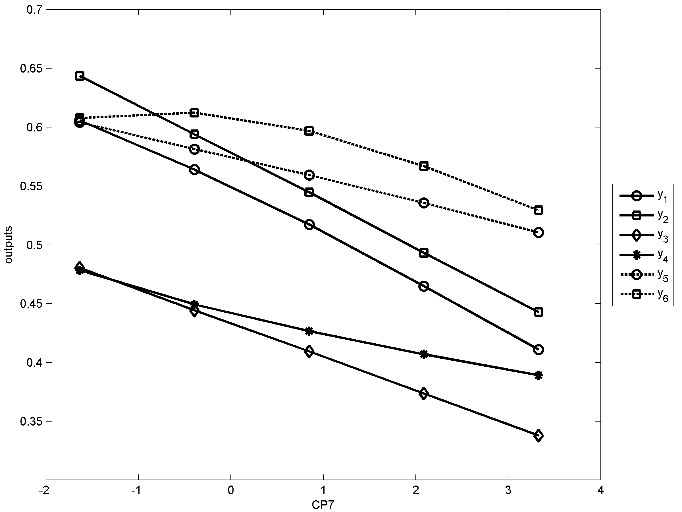

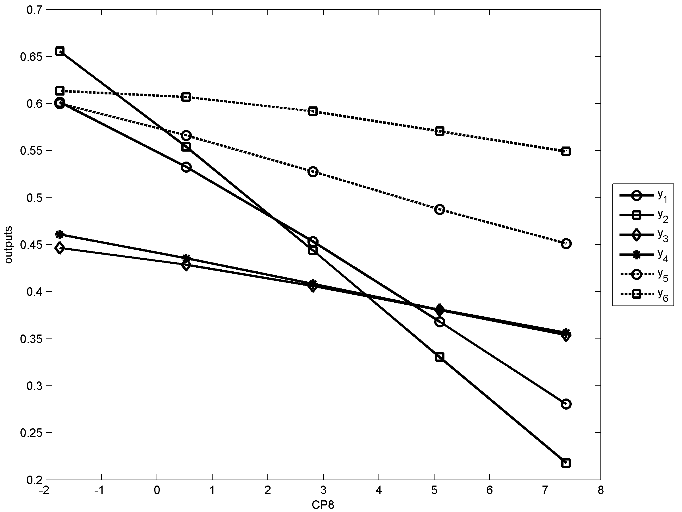

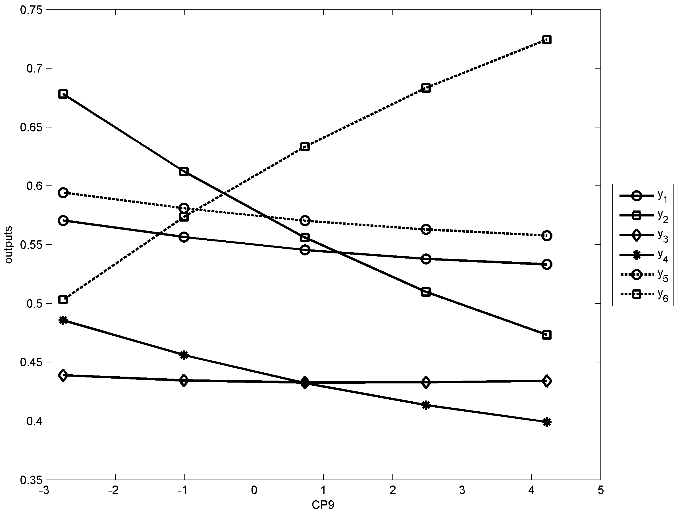

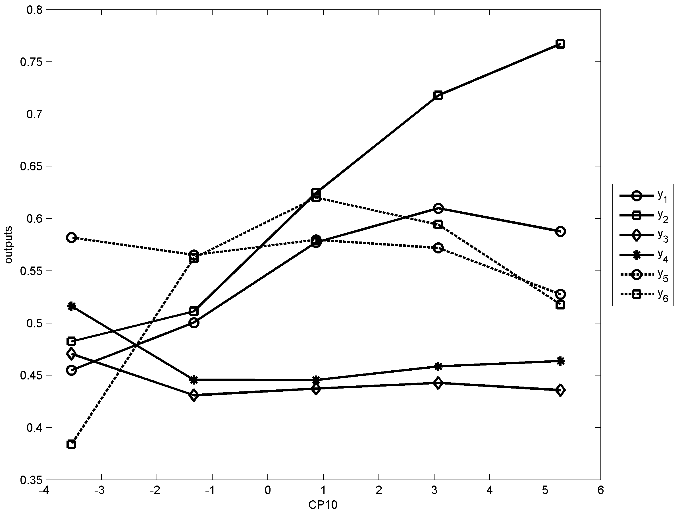

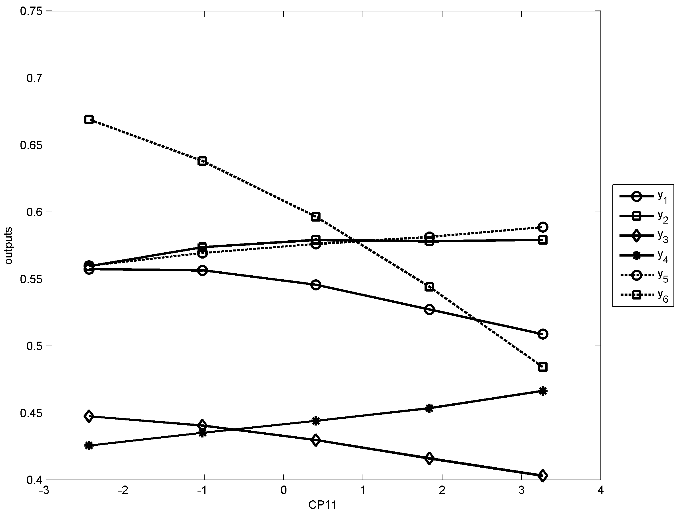

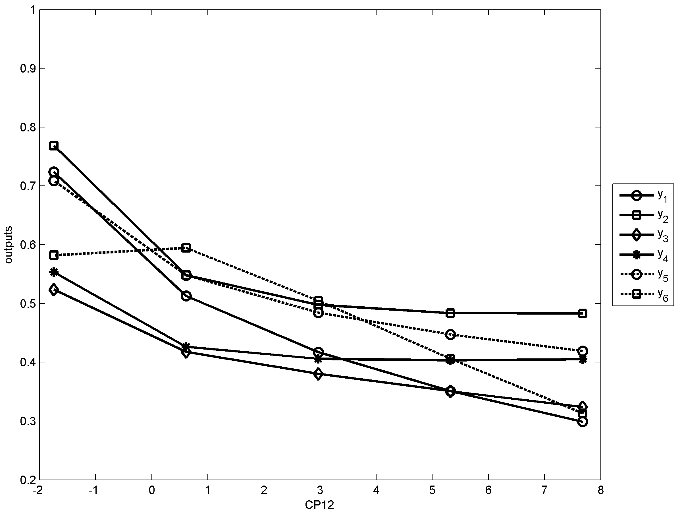

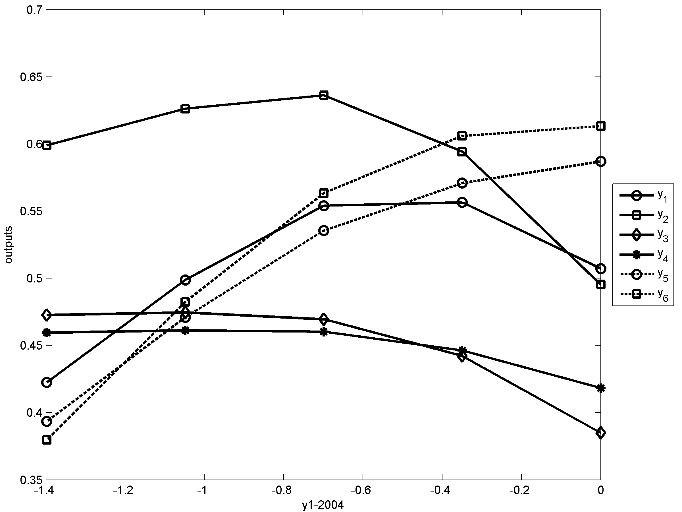

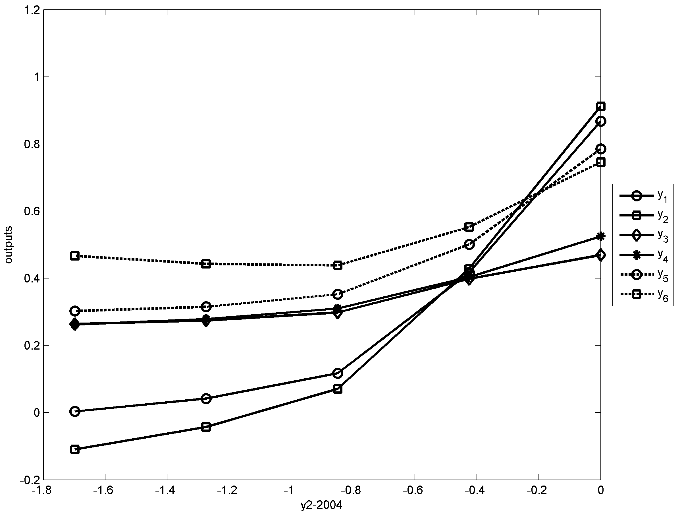

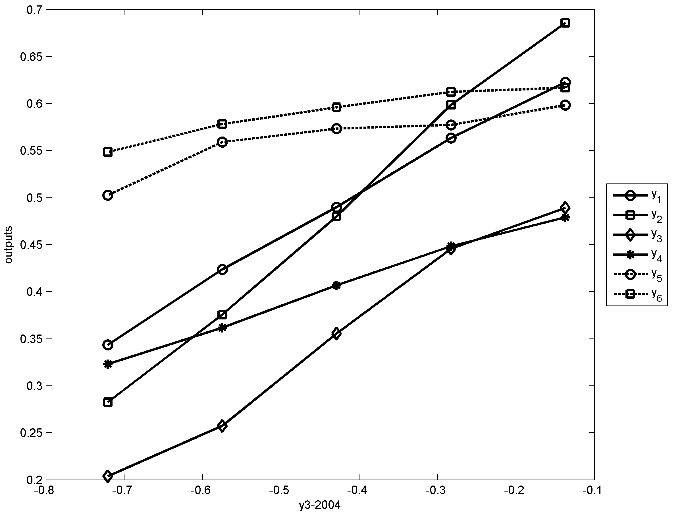

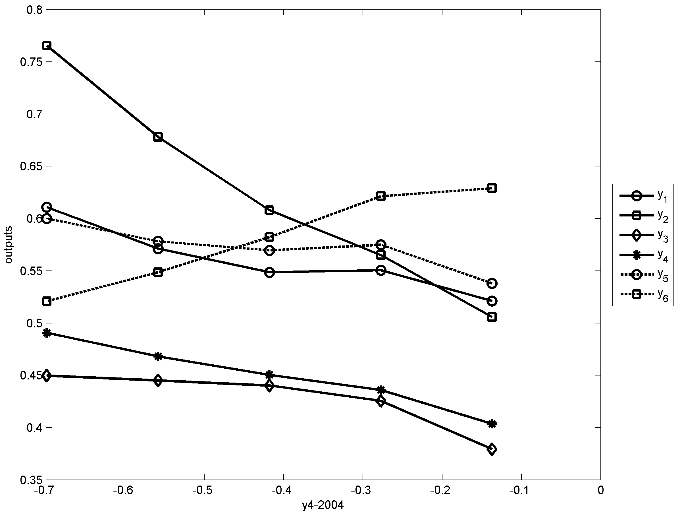

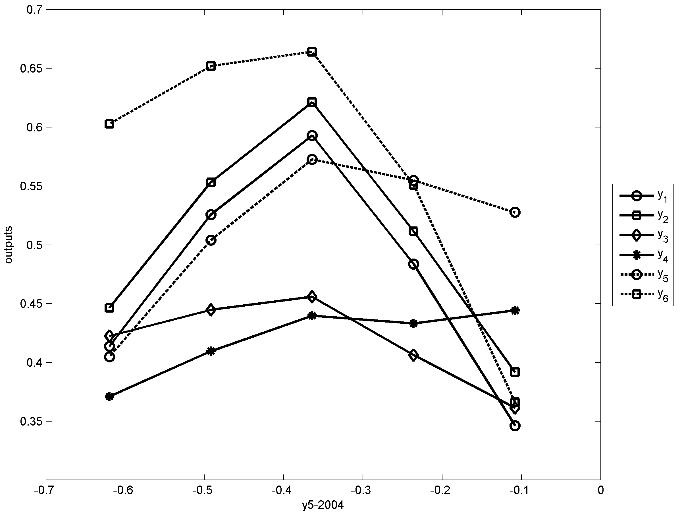

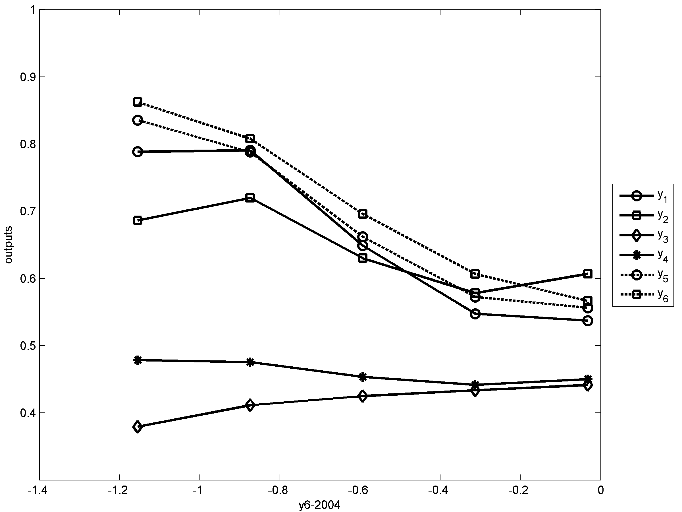

Supplement: S3 Appendix — (DOCX) [file pone.0185755.s003.docx]
